# High-Potential Hypervalent Antimony(V) Porphyrin–C_60_ Conjugates: Excitation Energy Transfer Dominates over Reductive Electron Transfer

**DOI:** 10.1021/acs.inorgchem.5c00294

**Published:** 2025-05-01

**Authors:** Niloofar Zarrabi, Jatan K. Sharma, Katya Andzelevich, Paul A. Karr, Art van der Est, Francis D’Souza, Prashanth K. Poddutoori

**Affiliations:** † Department of Chemistry & Biochemistry, University of Minnesota Duluth, 1038 University Drive, Duluth, Minnesota 55812, United States; ‡ Department of Chemistry, University of North Texas, 1155 Union Circle, # 305070, Denton, Texas 76203-5017, United States; § Department of Physical Sciences and Mathematics, Wayne State College, 1111 Main Street, Wayne, Nebraska 68787, United States; ∥ Department of Chemistry, Brock University, St. Catharines, Ontario L2S 3A1, Canada

## Abstract

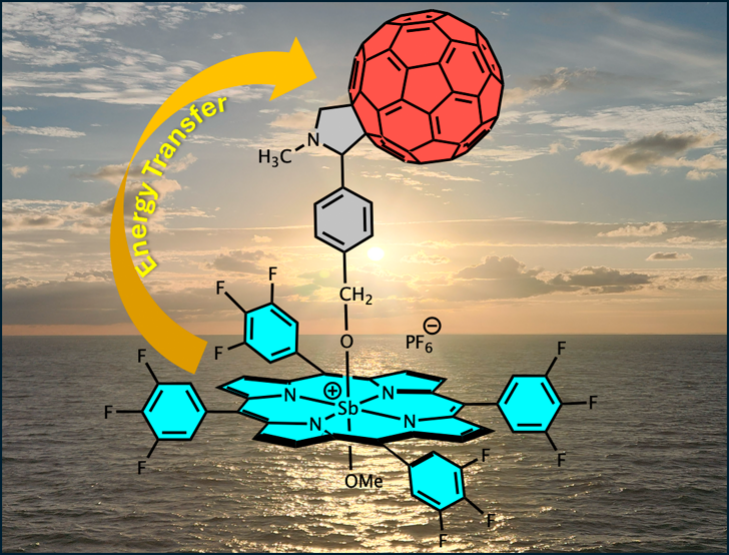

High-potential hypervalent antimony(V) porphyrins have been covalently linked to the well-known electron acceptor C_60_ molecule, resulting in the formation of antimony(V) porphyrin-fullerene conjugates: SbP–C_60_ and SbPF_3_–C_60_. The two porphyrins, SbP and SbPF_3_ contain four *meso*-phenyl and *meso*-3,4,5-trifluorophenyl units, respectively. These systems are designed to leverage the exceptionally high redox potentials of SbP and SbPF_3_ to create an unusual pattern of excited state energies in a porphyrin-fullerene conjugate, in which the energy of the (porphyrin)^•–^–C_60_^•+^ state lies between the porphyrin and fullerene excited singlet states. Time-resolved spectral data show that ultrafast singlet–singlet energy transfer from the porphyrin to the C_60_ unit occurs. The estimated energetics suggest that the ^1^C_60_* state could be populated from porphyrin excited singlet state by either the usual Förster mechanism or by electron transfer from the C_60_ unit to the excited SbP/SbPF_3_ moiety followed by charge recombination. However, spectral features associated with the charge-separated state are not observed, and the energy transfer rates calculated for the Förster mechanism are in reasonable agreement with the experimental values. Thus, direct energy transfer appears to be the dominant process in these novel dyads derived from high-potential antimony(V) porphyrins.

## Abstract 2

High-potential antimony(V) porphyrins are linked to the C_60_ molecule to generate antimony(V) porphyrin−fullerene conjugates. The steady-state and time-resolved studies show that the observed fluorescence quenching results from the singlet−singlet energy transfer from the porphyrin to the C_60_ unit and by the reductive electron transfer from the C_60_ unit to the antimony(V) porphyrin.

## Introduction

Fullerene (C_60_) is the most extensively studied molecule as an electron acceptor in donor–acceptor (D–A) systems, playing a crucial role in mimicking and understanding the photoinduced electron transfer process.^1−6^ Its truncated icosahedron structure leads to low reorganization energy, making C_60_ an efficient electron acceptor.^7^ Among the various D–A systems, porphyrin–C_60_ conjugates hold an exceptionally prominent position due to their complementary optical and redox properties.^8−19^ Because of the extended π-conjugation of their tetrapyrrole rings, the porphyrins absorb light effectively in the UV–vis spectral range, making them excellent light-harvesting antennas for various applications. Additionally, porphyrins are redox active, and their redox potentials can be easily tuned through structural modifications without significantly altering their optical properties.^20^ In contrast, while fullerenes are not very strong light absorbers, they are easily reduced. Thus, combining porphyrins with C_60_ has proven a successful strategy for designing D–A conjugates, which undergo efficient electron transfer to yield long-lived charge separation. This approach mimics the electron transfer process in photosynthetic models^21^ and molecular photonic and electronic devices.^22^

Among porphyrins, the main group 15 porphyrins, specifically phosphorus(V) porphyrins, and antimony(V) porphyrins, exhibit several advantageous properties, including high redox potentials, excellent singlet-state quantum yields, and the ability to form axial covalent bonds.^23−27^ These characteristics have been highlighted in the literature in reports on the development of diverse donor–acceptor (D–A) systems for investigating energy and electron transfer processes in fields such as artificial photosynthesis,^28−48^ photodynamic therapy,^49−53^ sensors,^54,55^ molecular electronics and photonics,^56−60^ electro- and photocatalysis,^61−63^ and nonlinear optics.^64−66^ Within group 15, antimony(V) porphyrins hold a unique position due to the substantial positive shift of their oxidation and reduction potentials by many hundreds of millivolts compared to other porphyrins. Their reduction midpoint potentials, for example, range from −0.08 to −0.36 V vs SCE,^23,67^ compared to phosphorus(V) and Zn(II) derivatives of tetraphenyl porphyrins, which have potentials of −0.53 and −1.48 V vs SCE, respectively.^25,68^ Moreover, their reduction potentials are higher than that of C_60_ (−0.69 V vs SCE).^14^ As a result, antimony(V) porphyrins could serve as robust two-dimensional electron acceptors in D–A systems rather than the usual role of the porphyrin as the electron donor. Reversing the order of the electron transfer could provide new insights in the design principles for building D–A systems with C_60_ and porphyrin units. For instance, this design strategy might allow the reorganization energy of the fullerene cation to be studied in the formation of the unusual radical pair, (porphyrin)^•–^–C_60_^•+^. A small number of related systems have been reported in which electron transfer from C_60_ to an acceptor was induced by Sc(III), xanthene dye, or porphyrin radical ions.^69−71^ Such studies are very useful for shedding light on the properties of radical pairs containing the rare C_60_^•+^ species and their potential application in artificial photosynthesis, and molecular electronics and photonics.

**Figure 1:**
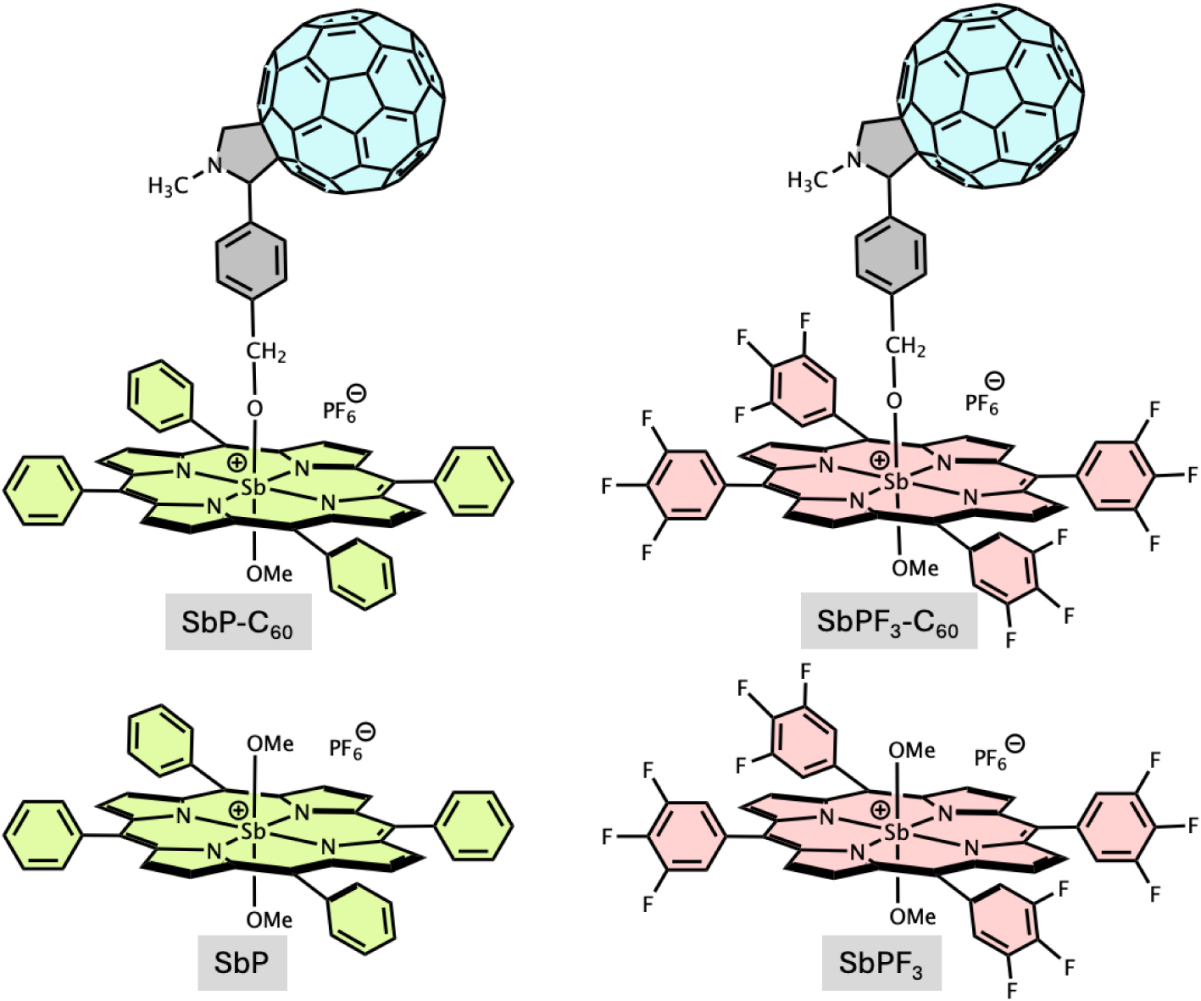
Studied “antimony(V) porphyrin–C_60_” conjugates and their reference monomer compounds.

The combination of antimony(V) porphyrin and C_60_ creates an intriguing situation in antimony(V) porphyrin–fullerene conjugates. Although C_60_ is an efficient electron acceptor, the exceptionally high oxidation potential of the porphyrin is expected to make the oxidative electron transfer from the porphyrin to C_60_ energetically unfavorable. On the other hand, the high reduction potential makes the reductive electron transfer, in which an electron is transferred from the C_60_ HOMO to the half-filled HOMO of the excited porphyrin, feasible. We will show that the estimated energy of the resulting (antimony(V) porphyrin)^•–^–C_60_^•+^ charge-separated state is higher than that of the excited singlet state of C_60_. This means that charge recombination could produce a fullerene excited singlet state by transferring an electron from the excited porphyrin LUMO to the fullerene LUMO. In addition, the thermodynamically feasible Förster energy transfer from the singlet excited porphyrin to the fullerene can also occur. This study aims to investigate whether these two processes occur and if they can be distinguished by their dependence on the properties of the porphyrin. To test this, we selected two versions of antimony(V) porphyrins: one with *meso*-phenyl groups and the other with *meso*-3,4,5-trifluorophenyl groups. The latter substitution will enhance the driving force for the anticipated reductive electron transfer in the proposed systems. To covalently bond the C_60_ moiety, we utilized the axial positions of the antimony(V) porphyrins. A unique aspect of antimony(V) porphyrins is their ability to accommodate two different axial substituents on opposite faces of the porphyrin.^46−48,51,55,60,62,72,73^ The structures of the new antimony(V) porphyrin–C_60_ conjugates are shown in Figure 1. Here, we evaluate their photophysical properties using steady-state and time-resolved spectroscopic techniques and taking into consideration both thermodynamic and kinetic factors. Our findings indicate that very efficient Förster energy transfer (EnT) from the antimony(V) porphyrins to the C_60_ unit is the main process that occurs. However, the dependence of the energy transfer rate on the solvent and on the redox potentials of the porphyrins can be rationalized as evidence for the possible involvement of an intermediate charge-separated state in addition to the direct resonant energy transfer pathway.

## Experimental Section

### Synthesis

The chemicals and solvents utilized in this study were purchased from Alfa Asear, Acros Organics Sigma-Aldrich, Tokyo Chemical Industry (TCI), or Fisher Chemical and were used as received. Anhydrous solvents were used in all the performed reactions. Chromatographic materials were purchased from SiliCycle or Sigma-Aldrich. The reaction schemes are represented in the Supporting Information: Scheme S1 depicts the synthesis of the C_60_–Tol and C_60_–Tol-Br, and Scheme S2 shows the reactions employed in the synthesis of SbP–C_60_ and SbPF_3_–C_60_. The precursor free-base porphyrins H_2_P and H_2_PF_3_, and the reference monomers SbP and SbPF_3_ were reported elsewhere.^23,67,74,75^ The SbP–C_60_ and SbPF_3_–C_60_ compounds were prepared as described in the following sections.

#### Synthesis of SbP–C_60_

SbP–OH (40 mg, 0.043 mmol), K_2_CO_3_ (7 mg, 0.051 mmol), and 18-crown-6 (7 mg, 0.027 mmol) were dissolved in 10 mL of dry toluene. The reaction flask was stirred for 5 min while purging with N_2_ at room temperature. At this stage, the C_60_–Tol-Br (26 mg, 0.027 mmol) was added and stirred at 60 °C under nitrogen atmosphere for 18 h. After this time the reaction was dried and redissolved in CH_3_OH, and the suspension was added NH_4_PF_6_ (100 mg, 0.61 mmol) to accomplish counterion exchange. The suspension was precipitated by adding water, then filtered to collect the precipitate and air-dried. The crude product was subjected to the column chromatography on silica gel. The column was eluted first with CH_2_Cl_2_ to remove the free-base porphyrin, C_60_–Tol-Br and SbP–OH and then eluted with CH_2_Cl_2_/EtOAc (=95:5) to obtain the product. The solvent was removed to yield the product in pure form. Yield = 41 mg (89%). ESI-MS: *m*/*z* 1646.2386 for [M – PF_6_]^+^, calcd 1646.2449 for C_115_H_43_N_5_O_2_Sb^+^. ^1^H NMR (CDCl_3_, 400 MHz): δ, ppm 9.42 (s, 8H), 8.25 (dd, 4H, *J* = 7.00 Hz), 8.19 (d, 4H, *J* = 7.00 Hz), 7.86 (m, 12H), 6.75 (bs, 2H), 4.73 (d, 1H, *J* = 9.9 Hz), 4.12 (s, 1H), 3.99 (d, 1H, *J* = 9.9 Hz), 3.90 (d, 2H, *J* = 7.4 Hz), 2.37 (s, 3H), −1.16 (d, 1H, *J* = 15.9 Hz), −1.36 (d, 1H, *J* = 15.9 Hz), −2.24 (s, 3H). ^31^P NMR (CDCl_3_, 162 MHz): δ, ppm −144.48 (sept, 1P, *J* = 715 Hz).

#### Synthesis of SbPF_3_–C_60_

The above similar procedure was utilized to obtain this compound: SbPF_3_–OH (47 mg, 0.041 mmol), K_2_CO_3_ (11 mg, 0.079 mmol), 18-crown-6 (7 mg, 0.027 mmol) were dissolved in 10 mL of dry toluene. The reaction flask was stirred for 5 min while purging with N_2_ at room temperature. To this, C_60_–Tol-Br (22 mg, 0.023 mmol) was added and the reaction stirred at 60 °C under nitrogen atmosphere for 24 h. After this time the reaction was dried and redissolved in CH_3_OH, and the suspension was added NH_4_PF_6_ (100 mg, 0.61 mmol) to accomplish counterion exchange. The crude was subjected to silica gel chromatography. The column was eluted first with CH_2_Cl_2_ to remove the free-base porphyrin, C_60_–Tol-Br and SbPF_3_–OH and then eluted with CH_2_Cl_2_/EtOAc (=95:5) to obtain the product. At this stage, minor impurities of C_60_–Tol-Br and SbPF_3_–OH were removed by washing with toluene followed by CH_3_OH to yield the product in pure form. Yield = 31 mg (67%). ESI-MS: *m*/*z* 1862.1342 for [M – PF_6_]^+^, calcd 1862.1319 for C_115_H_31_F_12_N_5_O_2_Sb^+^. ^1^H NMR (CD_3_CN, 400 MHz): δ, ppm 9.68 (s, 8H), 8.18 (bs, 4H), 6.85 (bs, 2H), 4.83 (d, 1H, *J* = 9.8 Hz), 4.57 (s, 1H), 4.08 (s, 3H), 2.43 (s, 3H), −1.22 (d, 1H, *J* = 14.0 Hz), −1.48 (d, 1H, *J* = 12.0 Hz), −2.26 (s, 3H). ^19^F NMR (CD_3_CN, 375 MHz): δ, ppm −72.9 (d, 6F, *J* = 706.0 Hz), −135.9 (bs, 1F), −136.4 (bs, 1F), −161.5 (bs, 1F). ^31^P NMR (CD_3_CN, 162 MHz): δ, ppm −144.65 (sept, 1P, *J* = 706 Hz).

## Results and Discussion

### Synthesis

Scheme S1 depicts the steps used to obtain the functionalized C_60_ molecules: C_60_–Tol-Br and C_60_–Tol. Scheme S2 summarizes the reactions leading to the target molecules. First, antimony(III) was inserted into the free-base porphyrins H_2_P or H_2_PF_3_ using SbBr_3_ to form the bromoantimony(III) porphyrins (Sb(III)PBr and Sb(III)PF_3_Br). In a later step, oxidation was performed using H_2_O_2_ in the presence of CH_3_OH to produce the asymmetrically substituted antimony(V) porphyrins (SbP–OH and SbPF_3_–OH) with axial −OH and −OCH_3_ units on opposite faces of the porphyrin plane. Counterion exchange was then used to produce the PF_6_^–^ salt. The available axial −OH group was utilized to attach C_60_ covalently through nucleophilic substitution with C_60_–Tol-Br to obtain the target axially substituted antimony(V) porphyrin-fullerene conjugates: SbP–C_60_ and SbPF_3_–C_60_. To avoid a possible mixture of counterions in the product, a second counterion exchange was performed with NH_4_PF_6_. The solid compounds were found to be stable for many months when stored in the air at room temperature. No degradation was observed under the experimental conditions used to characterize the photophysical properties.

### Structural Characterization

ESI mass spectrometry was used to carry out an initial characterization of the investigated compounds. For both SbP–C_60_ and SbPF_3_–C_60_ the mass spectra (Figures S1–S4) show an intense parent ion peak corresponding to the mass (*m*/*z*) of [M – PF_6_]^+^ with matching isotope distribution. The corresponding *m*/*z* values are given in the Experimental Section. The ^1^H and ^31^P NMR spectra of the investigated porphyrins and their respective monomer compounds are shown in Figures S5–S10. In the ^1^H spectra of SbP–C_60_ and SbPF_3_–C_60_ (Figures S9 and S10), shielding effects are apparent for the protons on the axial benzyloxy (−PhCH_2_O−) unit and are strongly shifted upfield due to the ring current effect of the porphyrin macrocycle. The protons from the phenyl and −CH_2_O– units appear around 7.81, 7.48, and 4.52 ppm in the free C_60_–Tol-Br shifts to 3.90, 6.75, and −1.16 and −1.36 ppm in the conjugate SbP–C_60_, 4.08, 6.85, −1.22 and −1.48 ppm in the conjugate SbPF_3_–C_60_, respectively. The two protons from the – CH_2_O– unit appear as two separate doublets due to their diasteriotopic nature. These upfield shifts confirm the formation of SbP–C_60_ and SbPF_3_–C_60_ conjugates. Interestingly, the *ortho*- and *meta*-protons of the *meso*-phenyl ring were split into two peaks in conjugates, SbPF_3_–C_60_ (Figure S9) and SbPF_3_–C_60_ (Figure S10), and their precursor porphyrins, SbP–OH (Figure S7) and SbPF_3_–OH (Figure S8). This splitting is attributed to the differing chemical environments on both faces of the porphyrin, i.e., one side holds an axial −OCH_3_ group, while the opposite side contains −OCH_2_PhC_60_/–OH. As a result, the ortho/meta-protons of the *meso*-phenyl ring experience different chemical environments, leading to slightly varied chemical shifts. Furthermore, these protons couple with neighboring fluorine atoms, evident in the ^1^H NMR spectrum of SbPF_3_–OH (Figure S8). The ^19^F NMR spectrum displays peaks corresponding to the counterion PF_6_^–^, as well as three fluorine atoms from the *meso*-phenyl rings. Notably, each fluorine on the phenyl rings presents a distinct peak, further indicating that the chemical environment differs on either side of the porphyrin plane. The ^19^F NMR also establishes the coupling between fluorine and phosphorus and fluorine and proton nucleus. The ^31^P NMR spectra of all the investigated compounds revealed five lines of the septet around −145.00 ppm from the PF_6_^–^ counterion.

### Absorption Studies

**Figure 2:**
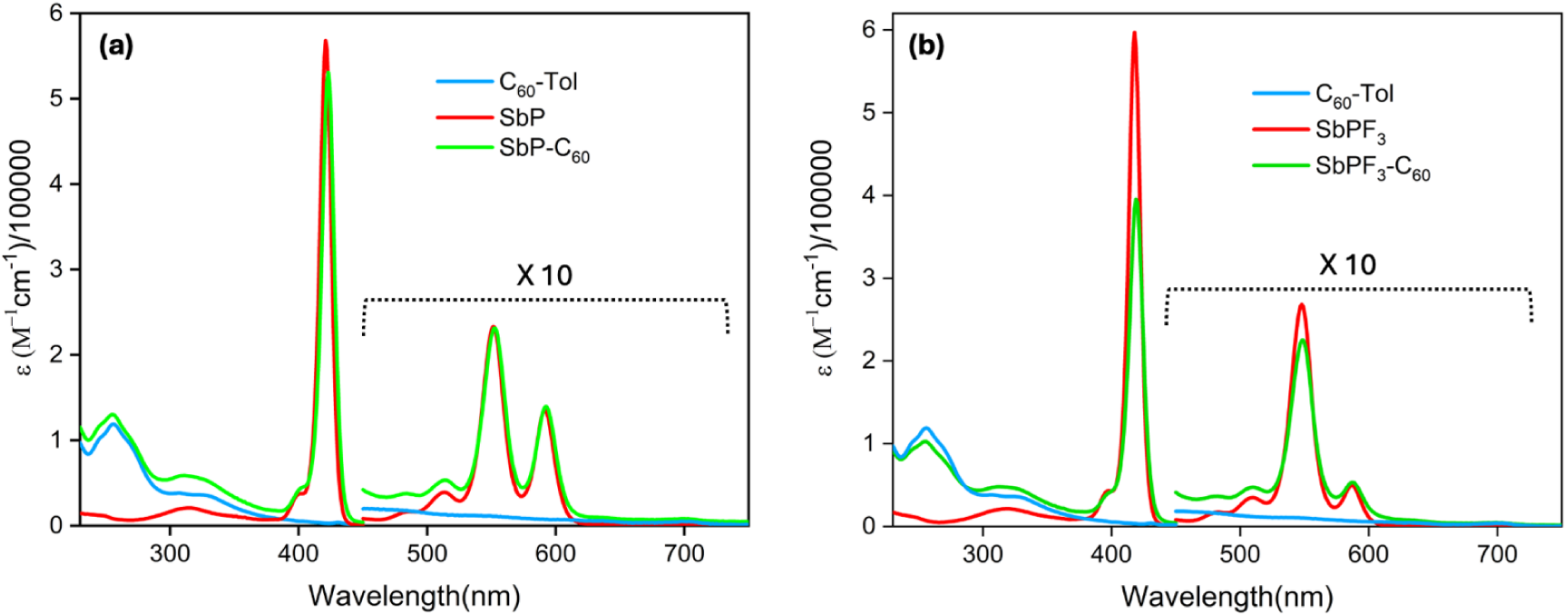
Electronic absorption spectra of (a) SbP–C_60_ and its reference compounds SbP and C_60_–Tol; (b) SbPF_3_–C_60_ and its reference compounds SbPF_3_ and C_60_–Tol in CH_2_Cl_2_.

The UV–visible spectra of antimony(V) porphyrins were measured in three solvents: CH_3_CN, CH_2_Cl_2_, and toluene. The spectra are presented in Figures 2 (in CH_2_Cl_2_), S11 (in toluene) and Figure S12 (in CH_3_CN), with the data (in CH_2_Cl_2_) summarized in Table 1. For comparison, reference porphyrins SbP, SbPF_3_, and C_60_–Tol are also reported. As illustrated in Figure 2, the reference porphyrins SbP and SbPF_3_ display a typical porphyrin spectrum, characterized by a strong B-band (Soret band) at 421 nm along with two weaker Q-bands at 552 and 591 nm. The C_60_–Tol reference compound shows pronounced π–π* absorption primarily in the ultraviolet region, featuring bands at 256 nm (ε = 5.08) and 318 nm (ε = 4.56). Additionally, a weak absorption is observed between 400 and 750 nm. The absorption spectrum of the SbP–C_60_ conjugate appears to be a superposition of the spectra of the individual chromophores, suggesting minimal or no perturbation of the ground-state electronic structures of the chromophores in this covalently linked formation. A similar trend is observed in the SbPF_3_–C_60_ conjugate, where its absorption spectrum (bands at 255, 318, 419, 549, and 587 nm) resembles a linear combination of the reference compounds SbPF_3_ (bands at 418, 549, and 586 nm) and C_60_–Tol (bands at 256 and 318 nm). The data were collected in the nonpolar solvent toluene (Figure S11) and the polar solvent CH_3_CN (Figure S12) to assess the solvent effect on the absorption spectra. In both cases, the absorption spectra remain similar to those of the reference compounds in their respective solvents.

**Table 1:** Absorption Data of Investigated Compounds in CH_2_Cl_2_

	UV–vis Absorption data λ, nm (log ε, cm^–1^ M^–1^)		
Sample	Q-band	S-Band	C_60_-band
**SbP–C**_**60**_	593 (4.14), 552 (4.37), 514 (3.71)	423 (5.72)	318 (4.76), 255 (5.12)
**SbP**	591 (4.11), 552 (4.35), 512 (3.58)	421 (5.75)	-
**C**_**60**_**–Tol**	-	-	318 (4.56), 256 (5.08)
**SbPF**_**3**_**–C**_**60**_	587 (3.71), 549 (4.34), 510 (3.67)	419 (5.60)	318 (4.68), 255 (5.01)
**SbPF**_**3**_	586 (3.73), 549 (4.43), 509 (3.59)	418 (5.78)	-

### Electrochemistry

**Figure 3:**
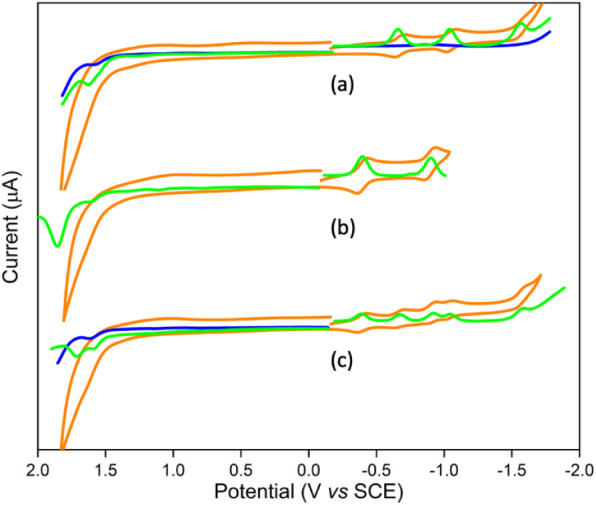
Cyclic (orange) and differential (green) voltammograms of (a) C_60_–Tol, (b) SbP, and (c) SbP–C_60_ with 0.1 M TBA·PF_6_ in CH_2_Cl_2_. The voltammogram of CH_2_Cl_2_ (blue) is also displayed for comparison purpose.

Cyclic and differential voltammograms of investigated conjugates were measured in CH_2_Cl_2_ with 0.1 M TBA·PF_6_. Representative voltammograms are shown in Figures 3 and S13, and the data are summarized in Table 2. The nature of the redox processes is established based on the peak-to-peak separation values (Δ*E*), and the cathodic-to-anodic peak current ratio. The voltammogram of reference porphyrin SbP revealed two reduction (−0.39 and −0.90 V) and one oxidation (1.86 V) processes under our experimental conditions. The reduction processes are found to be reversible, one-electron processes, whereas the oxidation process is an irreversible one-electron process. The C_60_ reference compounds show three reversible one-electron processes (−0.66, −1.04, −1.56) and one irreversible process (1.67 V). The SbP–C_60_ conjugate exhibits five reversible one-electron (−0.40, −0.67, −0.92, −1.04, and −1.58 V) and two irreversible (1.59 and 1.72 V) one-electron oxidation processes. By comparing the reference compounds, the first and third reversible processes are assigned to the reduction of the SbP unit, and the second, fourth, and fifth are assigned to the reduction of the C_60_ unit. The anodic scan reveals two oxidation processes (1.59 and 1.72 V) assigned to the oxidation of C_60_ and SbP units, respectively. These potentials are shifted slightly lower compared to the reference compounds, meaning that they are more easily oxidized when they are covalently linked. Importantly, the exceptionally strong electron withdrawing ability of Sb(V) pushes the first reduction potential of the porphyrin so positive that it becomes a better electron acceptor than the C_60_ unit.

**Table 2:** Absorption and Redox Data of Investigated Compounds in CH_2_Cl_2_ with 0.1 M TBA·PF_6_

	Redox data (V vs SCE)	
Sample	Oxidation	Reduction
**SbP–C**_**60**_	1.59, 1.72	–0.40, −0.67, −0.92, −1.04, −1.58
**SbP**	1.86	–0.39, −0.90
**C**_**60**_**–Tol**	1.67	–0.66, −1.04, −1.56
**SbPF**_**3**_	1.90	–0.15, −0.66
**SbPF**_**3**_**–C**_**60**_	1.68, 1.96	–0.15, −0.70, −1.09, −1.60

Similar results were found from the SbPF_3_–C_60_ conjugate (see Figure S13), where the redox processes are essentially a combination of its reference systems SbPF_3_ and C_60_, suggesting the absence of any electronic interaction between redox centers. However, electron-withdrawing fluorine on peripheral phenyls results in positively shifted potentials compared to the SbP–C_60_. The reduction of porphyrin becomes easier, so SbPF_3_ is an even better electron acceptor than the C_60_ unit. Similarly, the anodic scan of the SbPF_3_–C_60_ revealed that the oxidation potentials of C_60_ and SbPF_3_ at 1.68 and 1.96 V vs SCE (Figure S13) are more positive than those in the unfluorinated SbP–C_60_. This indicates that the SbPF_3_ porphyrin macrocycle is extremely electron-deficient and very hard to oxidize as a result of the combined effect of the highly electropositive Sb(V) center and the electron-withdrawing ability of the fluorine atoms on the phenyl substituents.

### DFT Calculations

**Figure 4:**
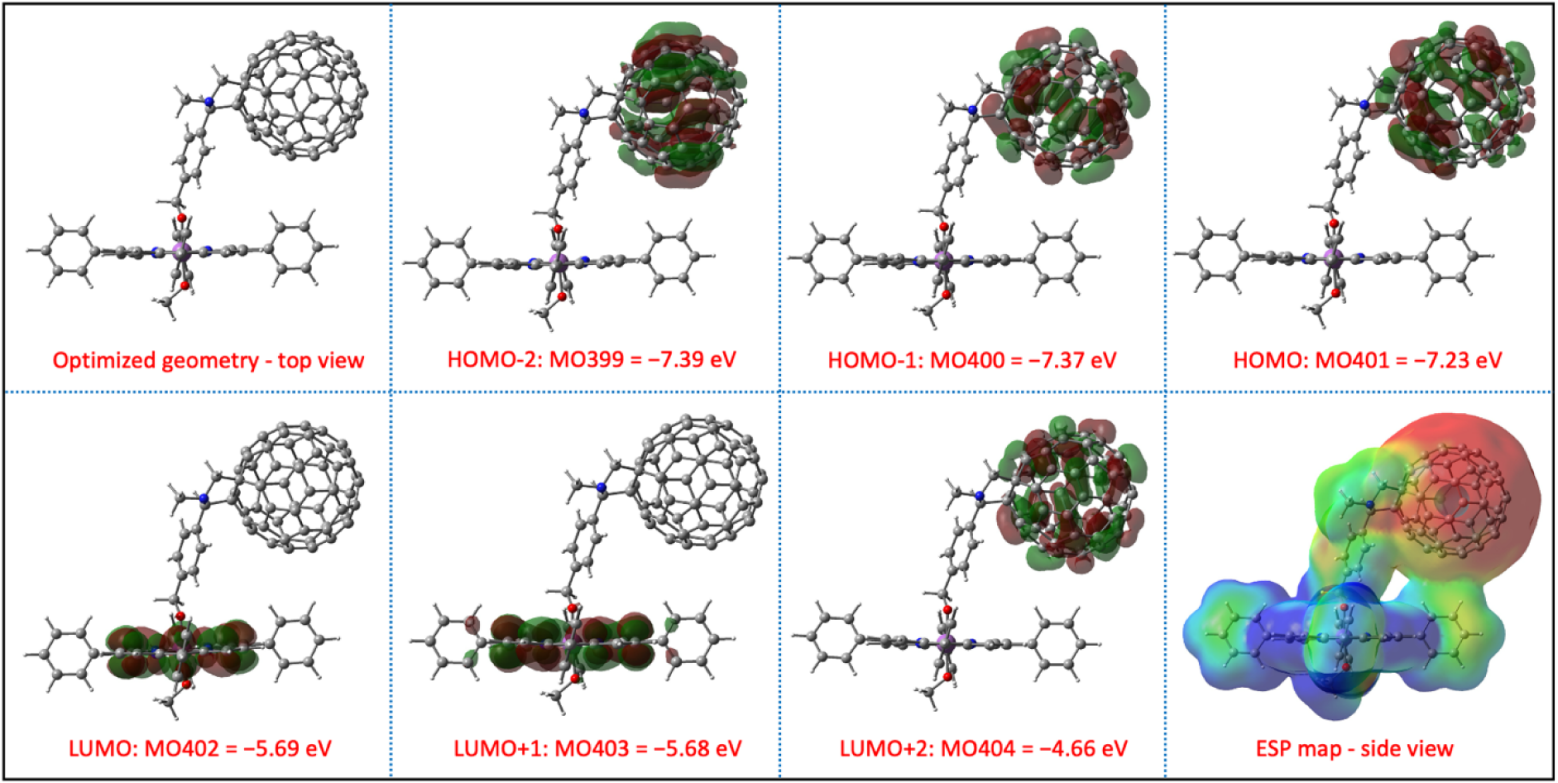
Structurally optimized SbP–C_60_ conjugate and their DFT calculated frontier molecular orbitals, energies and electrostatic potential maps. Blue = electron acceptor region. Red = electron donor region.

The geometry and electronic structures of the conjugates were predicted by performing Density Functional Theory (DFT) calculations. The structures were optimized to a stationary point on the Born–Oppenheimer potential energy surface using the B3LYP/GenECP model chemistry. The H, C, N, and O, atoms of the SbP–C_60_ molecule were modeled with the 6-311G(df,pd) basis and the Sb atom was modeled with the Def2TZVP effective core potential (ECP). The H, C, N, O, and F atoms of the SbPF_3_–C_60_ molecule were modeled with the 6-311G(df,pd) basis and the Sb atom was modeled with the Def2TZVP effective core potential (ECP), as parametrized in the Gaussian 16 software suite.^76^ As shown in Figure 4, the porphyrin and C_60_ units are well positioned in axial orientation with minimum or no π–π interactions between aromatic units. However, the frontier orbitals are quite interesting as the HOMO, HOMO – 1, and HOMO – 2 are exclusively localized on the C_60_ units, whereas the LUMO and LUMO + 1 are localized on the porphyrin. This is opposite to the situation in most porphyrin-fullerene conjugates.^14,16,18,19,77−79^ A similar orbital distribution is observed in SbPF_3_–C_60_; see Figure S14. The electrostatic potential maps (ESP) are shown in Figures 4 and S14, revealing electron-rich and deficient sites of the conjugates. This interesting distribution indicates the possible electron transfer from the C_60_ unit to the SbP/SbPF_3_. The DFT calculated HOMO–LUMO gap is found to be 1.54 and 1.29 eV in SbP–C_60_ and SbPF_3_–C_60_, respectively.

### Fluorescence Studies

**Figure 5:**
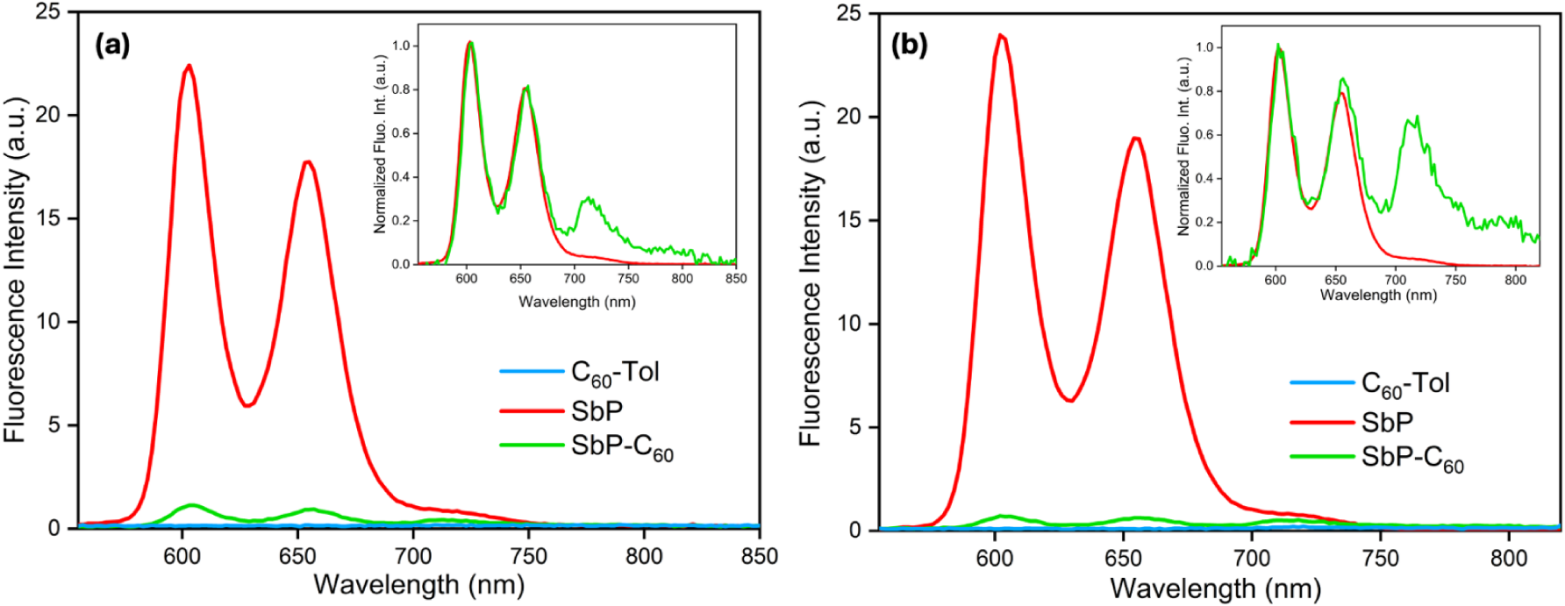
Fluorescence spectra of SbP–C_60_, SbP, and C_60_–Tol in CH_2_Cl_2_ at excitation (a) 545 nm, and (b) 410 nm. Inset shows the normalized fluorescence spectra of SbP–C_60_ and SbP.

**Figure 6:**
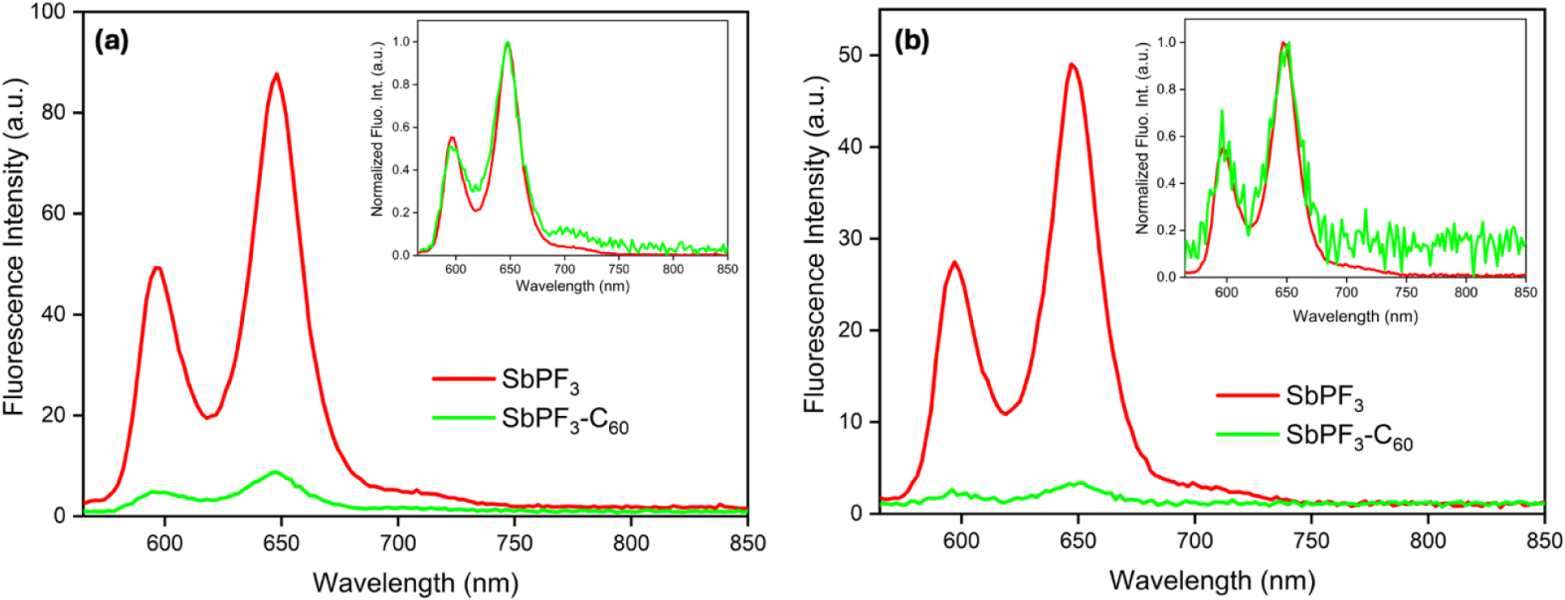
Fluorescence spectra of SbPF_3_–C_60_ and SbPF_3_ in CH_2_Cl_2_ at excitation (a) 557 nm, and (b) 424 nm. Inset shows the normalized fluorescence spectra of SbPF_3_–C_60_ and SbPF_3_.

The steady-state fluorescence spectra of the conjugates SbP–C_60_ and SbPF_3_–C_60_ and the reference compounds (SbP/SbPF_3_ and C_60_) measured with excitation at the Soret band and the Q-band absorption wavelengths in toluene (Figure S15), CH_2_Cl_2_ (Figures 5 and 6), and CH_3_CN (Figures S16 and S17). The corresponding data is summarized in Table 3. At these wavelengths, much of the absorption is due to the porphyrin. As seen in Figure 5, upon excitation of either the Soret band or the Q-band, the reference SbP and the conjugate SbP–C_60_ exhibit two emission bands located around 600 and 650 nm. However, the fluorescence of SbP–C_60_ is strongly quenched (%*Q* = ∼95%) compared to its reference compound SbP. Moreover, in the conjugate, two additional fluorescence bands are observed at ∼714 and 794 nm. Based on the fluorescence spectrum of the reference C_60_ compound (see Figure S18), these bands are assigned to the fluorescence of C_60_ unit in the conjugate. The normalized spectra in the inset clearly show these additional bands; see Figure 5 inset. Interestingly, such bands are very weak when C_60_ alone was excited at the same wavelength and concentration. Therefore, the observed fluorescence quenching suggests that the porphyrin excited state is depopulated by the energy transfer from porphyrin to C_60_, the electron transfer from C_60_ to SbP, or both. The very weak absorption of C_60_ (Figure 2, blue spectrum) occurs in the visible region, while the fluorescence bands of SbP (Figure 5) are in the red region of the visible spectrum. Hence, there is some spectral overlap between them, an essential feature for Förster energy transfer from SbP to C_60_. On the other hand, the energetics (discussed in the next section) suggest that SbP^•–^–C_60_^•+^ lies energetically below the excited singlet state of SbP with the free-energy change (Δ*G*_CS_) of −0.37 eV. Consequently, the strong quenching in the conjugate could also result from electron transfer. Similar results were found upon excitation of the Soret band. The fluorescence studies were also carried out in toluene (Figure S15) and CH_3_CN (Figure S16). However, the quenching does not depend strongly on solvent polarity, suggesting that either the electron transfer rate is not strongly solvent-dependent, or it only makes a minor contribution to the quenching.

**Table 3:** Fluorescence Data of Investigated Compounds

	Toluene		CH_2_Cl_2_		CH_3_CN	
Sample	λ, nm^a^ (%*Q*)	(τ, ns)^b^ (ChiSQ)	λ, nm^a^ (%*Q*)	(τ, ns)^b^ (ChiSQ)	λ, nm^a^ (%*Q*)	(τ, ns)^b^ (ChiSQ)
**C**_**60**_**–Tol**	718, 800	-	720, 800^c^	-	*insoluble*	
**SbP**	603, 655	1.04 (0.99)^23^	603, 655	1.24 (1.02)^23^	601, 653	1.36 (1.03)^23^
**SbP–C**_**60**_	605, 657, 717, 801 (94%)	*nd*	605, 655, 713, 789 (95%)	*nd*	601, 655, 715, 791 (95%)	*nd*
**SbPF**_**3**_	*insoluble*		596, 648	0.93 (1.09)	594, 646	0.99 (1.12)^67^
**SbPF**_**3**_**–C**_**60**_	*insoluble*		596, 648, 702 (91%)	*nd*	592, 646, 712, 800 (89%)	*nd*

a Fluorescence data, excitation at 330, 545, 555, or 557 nm. %*Q* is percent the quenching in porphyrin fluorescence. ChiSQ is the goodness of fit.

b Average lifetime data, excitation at 560 nm and emission at 600 nm. Lifetimes of the conjugates were within the time resolution of our setup, therefore, not determined (nd).

c In *o*-DCB.

Figure 6 depicts the fluorescence spectra of SbPF_3_–C_60_ and its corresponding reference SbPF_3_ at excitation 557 and 424 nm in CH_2_Cl_2_. Once again, two emission bands at 702 and 800 nm are observed, which are strongly quenched (%*Q* = ∼91%) in the conjugate. Interestingly, the C_60_ emissions are visible with ∼557 nm excitation but not with ∼420 nm. The observed C_60_ emission intensities are also lower than in the unfluorinated conjugate SbP–C_60_. This could be due to a lower yield of energy transfer to the ^1^C_60_* state or a decrease in its lifetime. Again, similar results were obtained in CH_3_CN, see Figure S17.

The excitation spectral studies of the conjugates and their reference monomers provide further evidence for the energy transfer process in all three solvents: toluene, CH_2_Cl_2_, and CH_3_CN. To ensure a fair comparison, the studies were conducted at constant concentrations, with excitation spectra collected at 800 nm, where only C_60_ emits. As illustrated in Figure S19, the excitation spectra of the SbP–C_60_ conjugate align closely with the absorption bands of SbP. Additionally, the intensity of the excitation spectrum for SbP–C_60_ is higher than that of the reference SbP (which lacks the axial C_60_ unit). This suggests that a significant number of porphyrin-excited photons lead to the emission of photons at 800 nm in SbP–C_60_. These findings indicate the presence of energy transfer from the ^1^SbP* to the C_60_ unit. Similar observations were noted in toluene and CH_3_CN, although the data is not shown here. In contrast, the excitation spectrum for SbPF_3_–C_60_ reveals the absorption peaks associated with the SbPF_3_ moiety, but this spectrum is considerably weaker than that of the SbP–C_60_ conjugate. The comparable intensities of SbPF_3_–C_60_ and SbPF_3_ indicate that energy transfer is less efficient in the SbPF_3_–C_60_ than in the SbP–C_60_ conjugate. However, steady-state fluorescence studies reveal that the %*Q* values for the two investigated conjugates are similar, suggesting that a reductive electron transfer may also serve as an additional decay pathway in the SbPF_3_–C_60_ conjugate.

Time-resolved fluorescence studies of conjugates and their reference monomers were measured, and such data is summarized in Table 3. The average value was considered for discussion purposes. The SbP and SbPF_3_ have lifetimes around ∼1.30 and ∼0.95 ns, respectively. However, the corresponding lifetimes in SbP–C_60_ and SbPF_3_–C_60_ were found to be much smaller and they are within the time resolution of the experimental setup.

### Energetics

**Figure 7:**
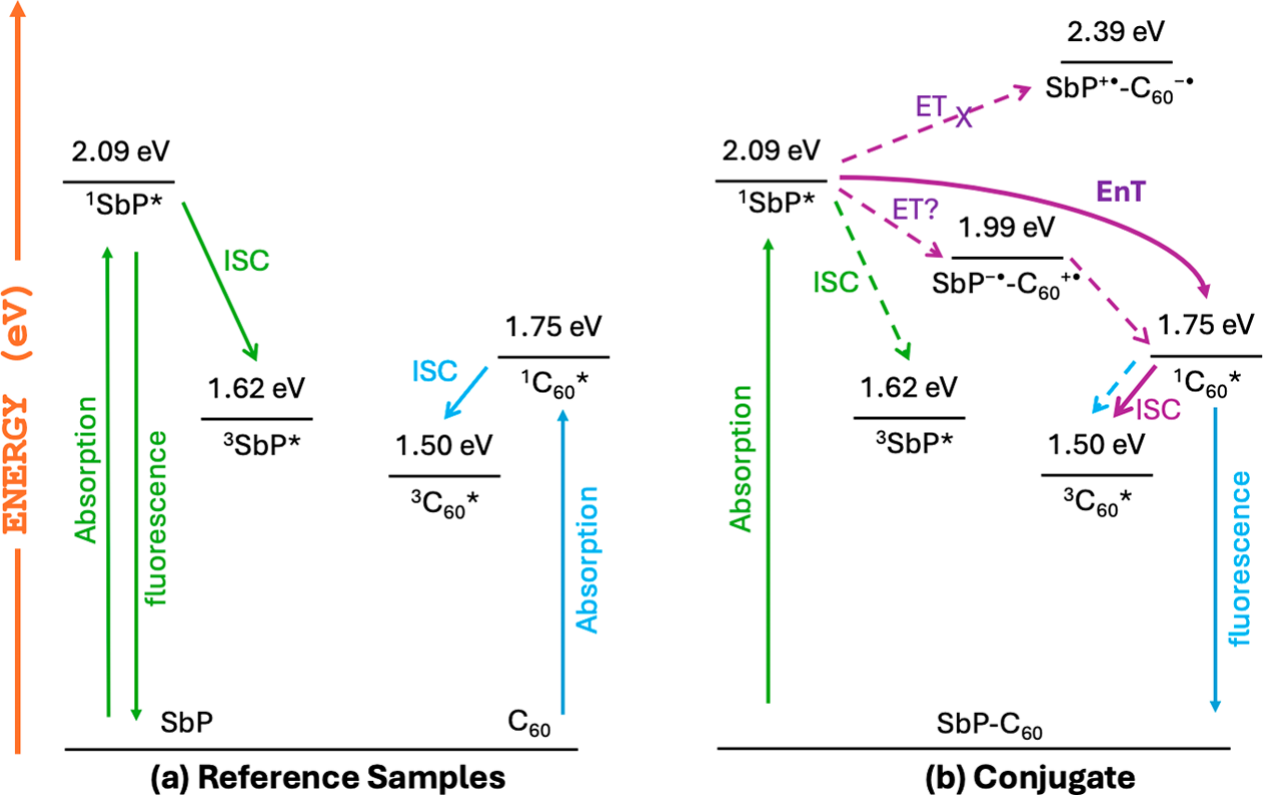
Energy level diagram of (a) reference compounds SbP and C_60_–Tol, and (b) conjugate SbP–C_60_ in CH_2_Cl_2_. Solid and dashed arrows represent directly detected and undetected processes, respectively.

The driving force for possible intramolecular energy and electron transfer processes can be estimated using the optical data and redox potentials of the components of the conjugates. The energy level diagram derived from these data is shown in Figures 7 and S20. The energies of the lowest excited singlet state (*E*_S_) of the SbP and SbPF_3_ were calculated from its optical absorption and emission spectra, see Figure S21. The singlet and triplet energies of C_60_;^80^ and triplet state energies of SbP and SbPF_3_ were adapted from literature.^23,67^The energies of the radical ion pair states and free energy change for charge separation are estimated using the equation: , where  is the first oxidation potential of the C_60_,  is the first reduction potential of the acceptor (SbP/SbPF_3_). The estimated Δ*G*_CS_ values found to be −0.10 eV and −0.29 eV for SbP–C_60_ and SbPF_3_–C_60_, respectively, suggesting the reductive electron transfer from C_60_ to SbP/SbPF_3_ is possible, and the rate could be somewhat faster in SbPF_3_–C_60_. The energies of these charge separated states are unusual in these conjugates. Many structurally similar porphyrin-fullerene conjugates have been reported in the literature using a variety of transition metals and main group elements in the porphyrin. In most cases, the (Porphyrin)^•+^–C_60_^•–^ state is lower in energy than the porphyrin and C_60_ excited singlet states, and the (Porphyrin)^•–^–C_60_^•+^ state is much higher in energy than these states. Here, the extreme electron-withdrawing ability of the SbP/SbPF_3_ reverses the order of the two charge-separated states and places the SbP^•–^–C_60_^•+^ (or SbPF_3_^•–^–C_60_^•+^) state between the porphyrin and C_60_ excited singlet states. This creates two possible pathways from the ^1^SbP* (or ^1^SbPF_3_*) state to the ^1^C_60_* state via resonant energy transfer or by charge separation and recombination. The observed fluorescence quenching shows that these are the main relaxation pathways in the conjugates. However, the competition between these two pathways depends on the thermodynamic properties (driving force) and the activation barrier to electron transfer versus the spectral overlap and distance between the chromophores, which govern resonant energy transfer. Thus, it is not immediately apparent which of them dominates.

### Time-Resolved EPR Studies

**Figure 8:**
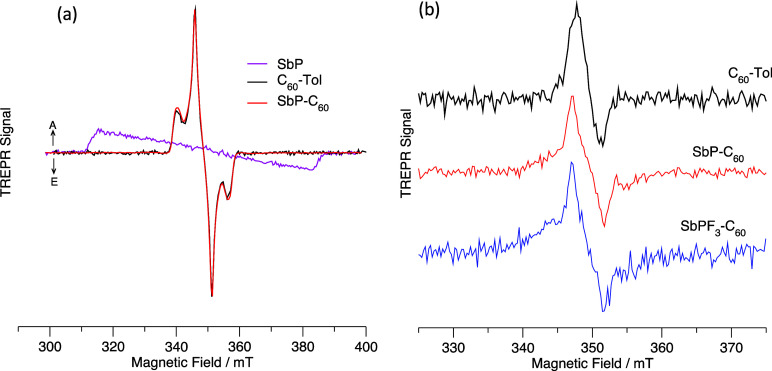
Spin-polarized transient EPR spectra of SbP, C_60_–Tol, SbP–C_60_, and SbPF_3_–C_60_. (a) glass phase spectra measured at 80 K in 2-MeTHF. (b) Room temperature spectra measured in the liquid crystal 5CB. The low-temperature spectra are the average signal intensity at a 700 ns wide time window centered at 1.75 μs after the laser flash. The room temperature spectra are the signal intensity in a 500 ns wide time window centered at 550 ns after the laser flash. The spectra are normalized to give approximately the same integrated intensity of the absorptive half of the spectrum.

The presence of light-induced triplet states and radical pairs can be detected using time-resolved EPR spectroscopy. The spin polarization patterns of these states also provide information about the pathway by which they were formed. Figure 8a shows transient EPR (TREPR) spectra of SbP–C_60_ and its two reference compounds C_60_–Tol and SbP measured at 80 K in 2-methyl THF. Corresponding spectra for SbPF_3_–C_60_ are shown in Figure S22 and are virtually identical. The spectra of the reference compounds are from their respective lowest excited triplet states formed by spin–orbit coupling mediated intersystem crossing (ISC). The spectrum of C_60_–Tol is much narrower than that of SbP because the average interspin distance is larger in the ^3^C_60_ state, and because its symmetry is higher. For SbP–C_60_ and SbPF_3_–C_60_, only the C_60_ triplet state is observed. Thus, we can conclude that the excited singlet state of the porphyrin is depopulated by another process which is much faster than ISC. At low temperatures in the glass phase, electron transfer is not expected because solvent reorganization, which is usually needed to stabilize the charge separation, cannot take place. Thus, the low-temperature TREPR spectra are consistent with resonant energy transfer to C_60_. Similar spectra taken at room temperature in the liquid crystal 4′-pentyl-4-biphenylcarbonitrile (5CB) are shown in Figure 8b. This solvent has been used because it partially aligns the molecules in the magnetic field. Without this alignment, the fast motion of the solutes averages the absorptive and emissive contributions, giving no net EPR signal. Liquid crystalline solvents are also known to stabilize charge-separated states.^15,81−83^ As with the low-temperature TREPR spectra, only the spectrum of the ^3^C_60_* state is observed in the conjugates, SbP–C_60_ and SbPF_3_–C_60_, and the spin polarization pattern indicates that it is formed by spin–orbit coupling mediated ISC from the ^1^C_60_* state. Hence, we can conclude that the porphyrin triplet state is not populated, any radical pair states that are formed are too short-lived to be observed directly by TREPR, and there is no evidence of radical pair recombination to either the porphyrin or C_60_ triplet states. Thus, room temperature TREPR spectra are consistent with singlet energy transfer from the porphyrin to C_60_. However, they do not allow the two possible pathways to be distinguished apart from the restriction that any radical pairs must have lifetimes less than the ∼100 ns rise time of the spectrometer.

### Transient Absorption Studies

**Figure 9:**
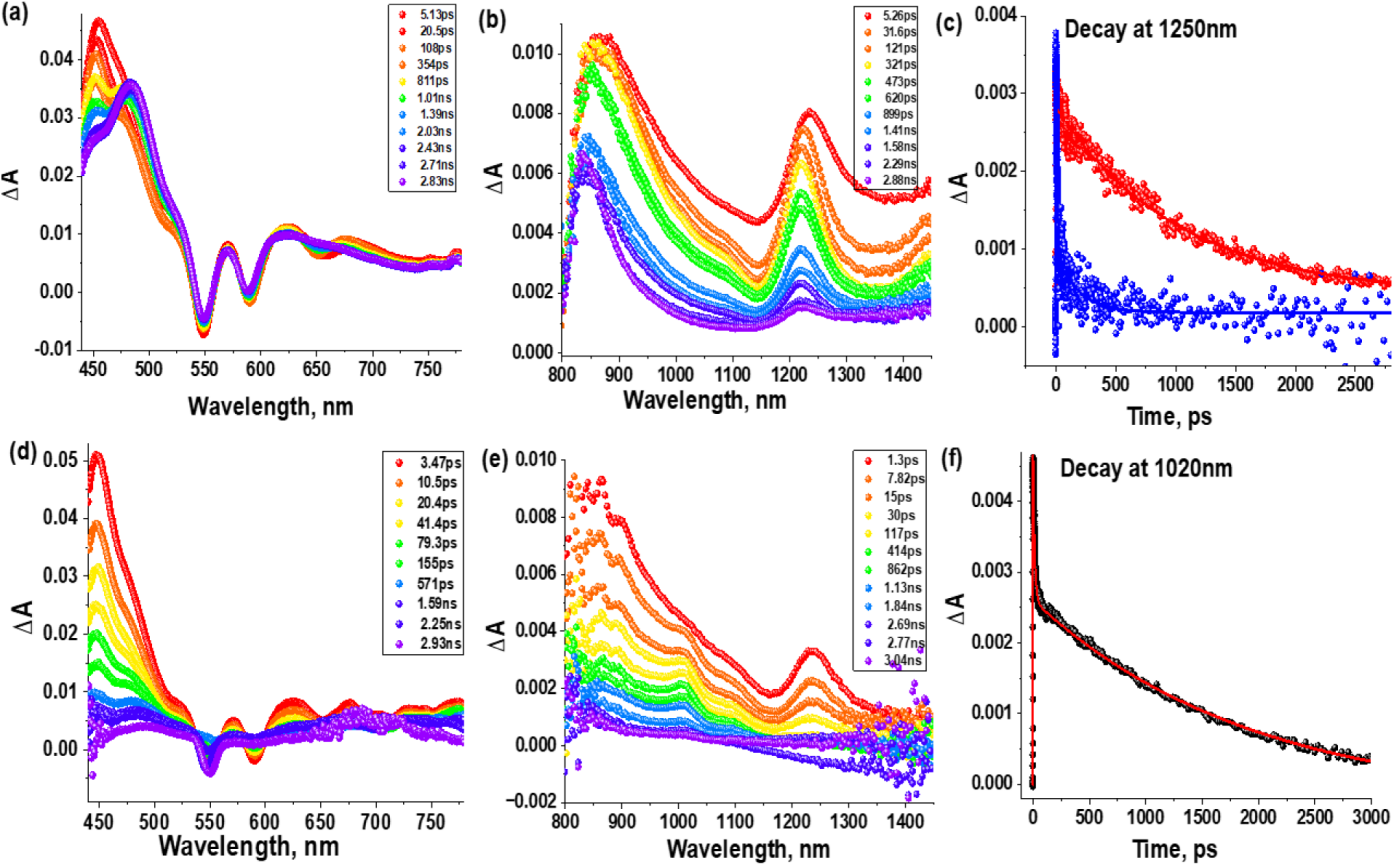
Femtosecond transient absorption spectra decay curves (a) SbP visible region; (b) SbP near-infrared region (NIR); (c) time profile of the 1250 nm band corresponding to the SbP (red) and SbP–C_60_ (blue); (d) SbP–C_60_ visible region and (e) SbP–C_60_ NIR region (f) decay profile of SbP–C_60_ at 1020 nm. The delay times of the spectra are indicated in the legends. The data were obtained in CH_3_CN with λ_exc_ = 550 nm.

**Figure 10:**
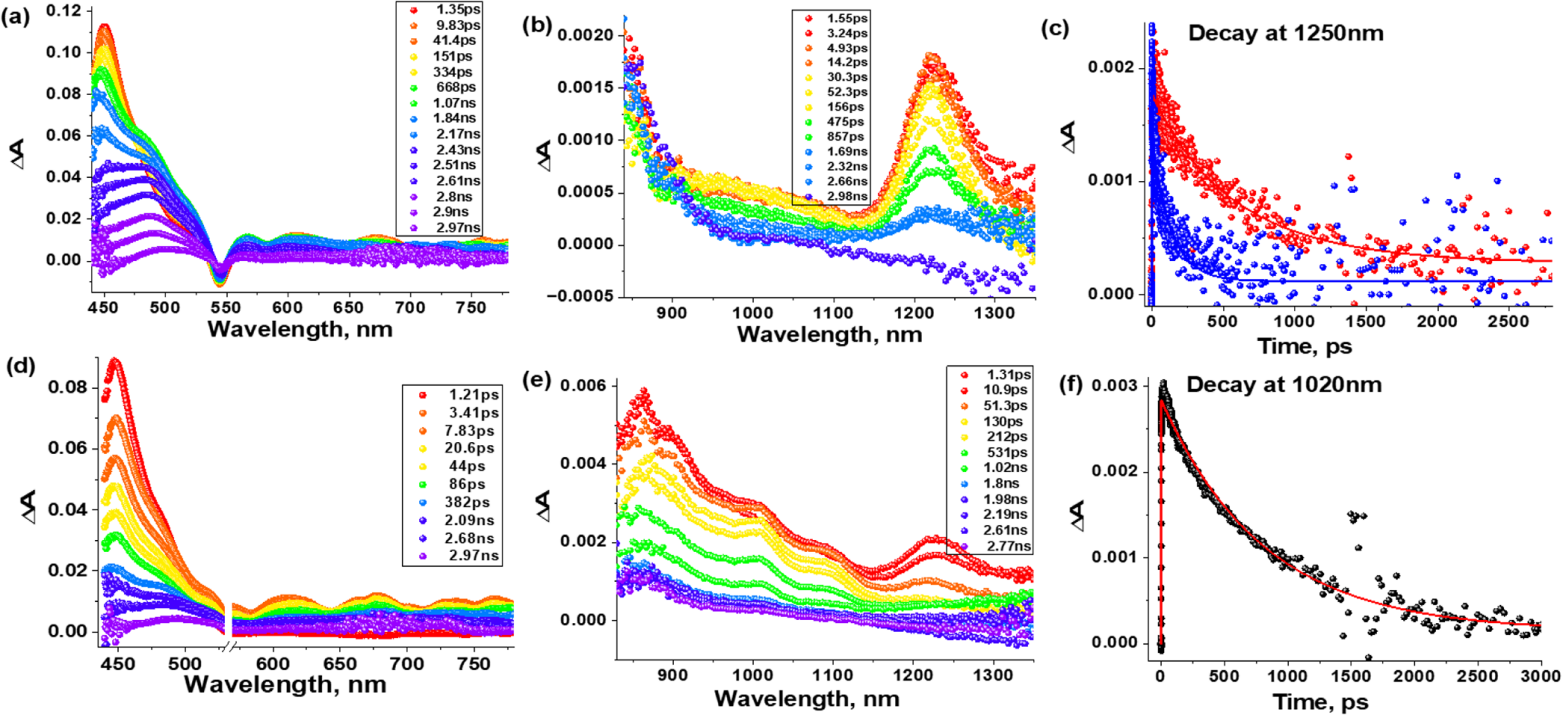
Femtosecond transient absorption spectra at indicated delay times (λ_exc_ = 550 nm) of (a, b) SbPF_3_ (visible and NIR region) and (d, e) SbPF_3_–C_60_ (visible and NIR region) in CH_3_CN. Right panel (c) shows the time profile at 1250 nm of SbPF_3_ (red) and conjugate SbPF_3_–C_60_ (blue), and (f) shows the decay profile at 1020 nm for the conjugate.

Femtosecond transient absorption (*fs*-TA) spectral studies were performed at excitation wavelength 550 nm to provide more direct experimental information about the relaxation pathways in SbP–C_60_ and SbPF_3_–C_60_. Figure 9 shows the *fs*-TA spectra at the indicated delay times for SbP and SbP–C_60_ in CH_3_CN. Corresponding *fs*-TA spectra of C_60_–Tol in benzonitrile are shown in Figure S23. For the conjugate and reference porphyrin, the instantaneously formed porphyrin singlet excited state has positive peaks at 445, 570, 625, 870, and 1250 nm due to the excited state absorption (ESA) (Figure 9a,b, red spectra). Negative peaks are also observed at 550 and 590 nm that correspond to ground-state bleaching (GSB) of the Q-bands (compare with Figure 2a). The peak at 590 nm also contains a contribution from stimulated emission (SE). In the visible region data set for SbP (Figure 9a), the peak at 445 then decays because of the ISC, and a new peak at 485 nm due to ^3^SbP* rises, while the negative GSB bands decay only slightly. In the IR region (Figure 9b), the bands at 870 and 1250 nm also decay with a time constant of ∼1 ns as ISC occurs. In the case of the conjugate, additional peaks at early time due to the ESA of C_60_ are expected at 500, 761, 886, and 1020 nm, as seen in the reference spectrum of C_60_–Tol (Figure S23). However, these absorbance changes are much weaker than those of the porphyrin. In contrast to the SbP reference, the transient spectra of the SbP–C_60_ at 2–3 ns (Figure 9d,e, blue and purple spectra), there is almost no ESA from the porphyrin. This is consistent with the strong fluorescence quenching and shows that the porphyrin-excited singlet state decays by energy or electron transfer before intersystem crossing occurs in the conjugate. In the IR region (Figure 9e), a weak peak at 1020 nm, corresponding to ^1^C_60_* ESA (see Figure S23), grows as the peak at 1250 nm due to ^1^SbP* ESA decays, providing evidence for energy transfer from ^1^SbP* to C_60_ to produce ^1^C_60_*. The decay profile of the 1020 nm peak is shown in Figure 9f. Since the ESA peaks at 1020 nm from ^1^C_60_* and 1250 nm from ^1^SbP* are far from other transient bands, they can be used to estimate the rate constant of the energy transfer *k*_EnT_. The decay profile of SbP at 1250 nm (Figure 9c, red curve) has a lifetime of ∼1.5 ns, which is governed primarily by ISC from ^1^SbP* to ^3^SbP*. The corresponding curve of the conjugate (Figure 9c, blue curve) is biexponential, where the first fast component accounts for almost all of the absorbance change corresponding to EnT, whereas the second minor component represents the ^1^SbP* lifetime in a small fraction of molecules in which EnT does not occur (see Table 4). The population of ^3^C_60_* via intersystem crossing of ^1^C_60_* at longer delay times is also witnessed, as shown by a broad peak in the 700 nm range^84^ at later delay times (see Figure 9d). It is also worth noting that in both conjugates, the formation of ^3^C_60_* at later delay times was apparent, with a broad peak in the 700 nm ranges (see Figures 9d, 10d, S25d and S26c), confirming the conversion of ^1^C_60_* to ^3^C_60_* via the process of intersystem crossing. These *fs*-TA experiments were also performed in the less polar solvent toluene (see Figure S25), which shows longer triplet state lifetimes and slightly faster energy transfer rates *k*_EnT_ than in CH_3_CN. To obtain the triplet state lifetimes, nanosecond transient absorption (ns-TA) data were recorded on a longer time scale (>3 ns) (Figure S24). While both SbP and SbPF_3_ had features characteristics of ^3^SbP* (strong transient signal in the 440–550 nm range with triplet lifetimes of 3.4 and 4.2 μs, respectively, for SbP and SbPF_3_ in acetonitrile), for the donor–acceptor conjugates, the prominent peak was centered around 700 nm, corresponding to ^3^C_60_* (Figure S24). The measured lifetimes for ^3^C_60_* were 3.6 and 3.4 μs (see Figure S24 insets for decay curves).

**Table 4:** Rate Constants for Energy Transfer *k*_EnT_, Triplet State *k*_T_, and Triplet State Lifetime τ_T_ from Femto- and Nanosecond Transient Absorption Spectroscopy for the Investigated Samples

Sample	Solvent	Lifetimes^a^ (ps)	*k*_EnT_ × 10^10^ (s^–1^) Expt.^b^	*k*_EnT_ × 10^10^ (s^–1^) Calcd	*k*_T_ × 10^6^ (s^–1^)	τ_T_ (in μs)
**SbP**	Toluene	841.9(100)	-	-	0.09	10.41^c^
	CH_3_CN	1.9(15.7), 1068.2(84.3)	-	-	0.29	3.41^c^
**SbP–C**_**60**_	Toluene	8.6(62.8), 31.4(37.2)	11.6	1.5	0.64	1.56^d^
	CH_3_CN	10.3(78.2), 169.5(21.8)	9.7	2.0	0.29	3.36^d^
**SbPF**_**3**_	Toluene	insoluble	-	-	-	-
	CH_3_CN	599.9(100)	-	-	0.23	4.29^c^
**SbPF**_**3**_**–C**_**60**_	Toluene	-	20.6	-	0.40	2.46^d^
	CH_3_CN	5.2(64.7), 133.6(35.3)	19.2	2.2	0.31	3.24^d^

a Decay lifetime for 1250 nm peak = τ_1_ ps (%Amplitude) and τ_2_ ps (%Amplitude).

b Estimated error = ±10%. The Φ and τ of SbPF_3_ are not know in toluene due to insolubility, hence, the ***k*_EnT_** was not calculated for SbPF_3_–C_60_ in toluene.

c ^3^SbP* state.

d ^3^C_60_* state.

Figure 10 shows the *fs*-TA spectra of SbPF_3_ and SbPF_3_–C_60_ in CH_3_CN. The peak positions and their decay behavior are analogous to the unfluorinated compounds. However, as shown in Table 4, the energy transfer rate constant *k*_EnT_ in SbPF_3_–C_60_ is roughly a factor of 2 higher than in SbP–C_60_. In the case of CH_3_CN solvent, the rate constant *k*_EnT_ obtained from the faster decay component of the 1250 nm peak and the *k*_EnT_ obtained from the rise time of the 1020 nm peak that also corresponds to energy transfer, was found to be similar. For this reason, the rise time of 1020 nm peak was utilized to obtain *k*_EnT_ in toluene as 1250 nm ESA peak was weak in this solvent due to low molar absorptivity (see Figure S26d).

The energy transfer rates were also estimated based on the Förster dipole–dipole energy transfer mechanism given by
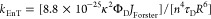
1where *n* is the solvent refractive index, Φ_D_ and τ_D_ are the fluorescence quantum yield, and the fluorescence lifetime of the reference donor (SbP or SbPF_3_), *J*_Forster_ is the spectral overlap integral between the emission of the donor and absorption of the C_60_ acceptor, and *R* is the donor–acceptor center-to-center distance (11.35 and 10.20 Å for SbP–C_60_ and SbF_3_–C_60_, from DFT optimized structures, respectively). The Φ_D_ and τ_D_ values for SbP and SbF_3_ were taken from the literature.^23,67^ The parameter κ^2^ is determined by the relative orientation of the transition dipoles of the donor and acceptor units, and its value ranges from 0 to 4. Considering the flexible nature of the connecting axial bond, a random distribution of dipole orientations was assumed and the corresponding value of κ^2^ = 2/3 was used.^85^ The PhotochemCAD program^86^ was employed to calculate the spectral overlap integral, *J*_Forster_ and the Förster energy transfer (*k*_EnT_) rates. The obtained rates are summarized in Table 4 and agree with the experimental values to within an order of magnitude. The fact that the calculated rates are lower than the experimental ones is likely due to the uncertainty in the orientation parameter κ^2^.

## Conclusions

In addition to their unusual redox properties, the compounds reported here illustrate how the asymmetric “axial-bonding” ability of antimony(V) porphyrins can be utilized to construct donor–acceptor conjugates. The synthesis protocols for producing such complexes are straightforward, using facile inorganic and organic reactions. The exceptionally high redox potentials of the antimony(V) porphyrins mean that the roles of the porphyrin are fullerene are reversed with the porphyrin being the better electron acceptor so that C_60_ might function as an electron donor. Density Functional Theory (DFT) and energetic calculations based on the redox potentials and optical properties indicate that electron transfer from the C_60_ unit to the excited antimony(V) porphyrin followed by recombination to ^1^C_60_* is energetically feasible. However, this pathway competes with resonant energy transfer from the singlet excited states of the antimony(V) porphyrins to the C_60_ unit. In agreement with these predictions, the steady-state fluorescence studies demonstrate significant quenching of the porphyrin-excited singlet state, and the transient EPR spectra show that the porphyrin triplet state is not populated. The transient absorbance studies indicate that quenching is mainly due to resonant energy transfer, which is supported by the agreement between the experimentally measured and theoretically estimated rates based on the Förster mechanism. The slightly higher rate of energy transfer in the SbPF_3_–C_60_ conjugate compared to SbP–C_60_ and the slightly lower values of the calculated rates both suggest the possible presence of a small amount of ultrafast electron transfer followed by charge recombination to populate ^1^C_60_*. However, these small differences in the rates lie well within the uncertainty in the calculated values, so it is only possible to conclude that any contribution from the electron transfer pathway must be small. Thus, the results show that in these novel conjugates the very high oxidation potential of the Sb porphyrin leads to resonant energy transfer being the dominant process.

## Supporting Information Available

Synthesis details, experimental methods, ESI mass spectra, NMR spectra, absorption spectra, cyclic voltammograms, DFT data, fluorescence spectra, energy level diagram, overlap of absorption and fluorescence spectra, TREPR spectra, and transient absorption spectra (PDF)
