# Supplementary material for: High-Potential Hypervalent Antimony(V) Porphyrin–C60 Conjugates: Excitation Energy Transfer Dominates over Reductive Electron Transfer
Source: Inorg Chem. 2025 May 1;64(18):8958–70. doi: 10.1021/acs.inorgchem.5c00294 (PMC12076542; doi:10.1021/acs.inorgchem.5c00294)
Supplement: Supplementary file 1 — ic5c00294_si_001.pdf [file ic5c00294_si_001.pdf]

Supporting Information

High-Potential Hypervalent Antimony(V) Porphyrin-C_{60} Conjugates: Excitation Energy Transfer Dominates over Reductive Electron Transfer

Niloofar Zarrabi,[a] Jatan K. Sharma,[b] Katya Andzelevich,[a] Paul A. Karr,[c] Art van der Est,[d] Francis D'Souza,*[b] Prashanth K. Poddutoori*[a]*

[a]Department of Chemistry & Biochemistry, University of Minnesota Duluth, 1038 University Drive, Duluth, Minnesota 55812, USA. Email: ppk@d.umn.edu

[b]Department of Chemistry, University of North Texas, 1155 Union Circle, # 305070, Denton, Texas 76203-5017, USA. Email: francis.dsouza@unt.edu

[c]Department of Physical Sciences and Mathematics, Wayne State College, 1111 Main Street, Wayne, Nebraska 68787, USA.

[d]Department of Chemistry, Brock University, St. Catharines, ON L2S 3A1, Canada. Email: avde@brocku.ca

Experimental section

Synthesis of C_{60}-Tol-Br. A mixture of C_{60} (100 mg, 0.14 mmol), sarcosine (26 mg, 0.28 mmol) and OHC-Ph-CH_2Br (55 mg, 0.28 mmol) in dry toluene (100 mL) was refluxed under nitrogen for 7 h. After reaction time, toluene volume reduced to 20 mL using rotavapor. Then solid pack was made of it using silica gel. The silica gel column was eluted with toluene: hexanes (=50:50). The desired compound collected as the second band. The solvent was evaporated to get the pure compound as a brown solid. Yield = 60 mg (46%).^{1}H NMR (400 MHz, $CDCl_3$): *δ, ppm* 7.81 (bs, 2H), 7.46 (d, 2H, J = 8.3 Hz), 5.01 (d, 1H, J = 9.8 Hz), 4.97 (s, 1H), 4.52 (s, 2H), 4.28 (d, 1H, J = 9.8 Hz), 2.83 (s, 3H).

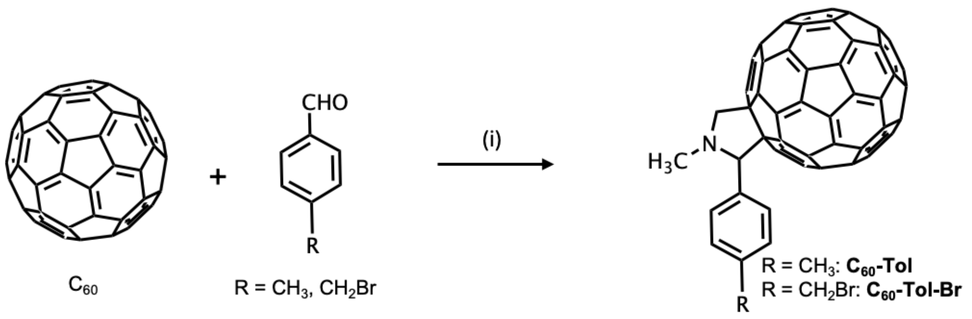

Scheme S1. Synthesis of fullerene derivative C_{60}-Tol and C_{60}-Tol-Br. *Reaction conditions*: (i) Sarcosine, aldehyde (tolualdehyde or 4-(bromomethyl)benzaldehyde), dry toluene, reflux under N_2.

Synthesis of C_{60}-Tol. A mixture of C_{60} (100 mg, 0.14 mmol), sarcosine (26 mg, 0.28 mmol) and OHC-Ph-CH_3 (34 mg, 33 µl, 0.28 mmol) in dry toluene (100 mL) was refluxed under nitrogen for 19 h. After reaction time, toluene volume reduced to 20 mL using rotavapor. Then solid pack was made of it using silica gel. The silica gel column was eluted with toluene:hexanes (=50:50). Desired compound collected as the second band. The solvent was evaporated to get the pure compound as a brown solid. Yield = 55 mg (46%). ESI-MS: *m/z* 868.1324.1520 for $[M +H]^+$, calcd 868.1121 for $C_{70}H_{14}N^+$. ^1H NMR (400 MHz, $CDCl_3$): *δ, ppm* 7.61 (bs, 2H), 7.16 (d, 2H, *J* = 8.0 Hz), 4.91 (d, 1H, *J* = 12 Hz), 4.83 (s, 1H), 4.18 (d, 1H, *J* = 8 Hz), 2.73 (s, 3H), 2.28 (s, 3H).

Synthesis of SbP-OH. H_2P (500 mg, 0.81 mmol) and $SbBr_3$ (1.20 g, 3.32 mmol) were heated under reflux for 3 days under N_2 in a solution containing 20 mL of dry CH_2Cl_2 and 0.8 mL of 2,6-Lutidine. After this time 2 mL of water and 2 mL of hexanes were added to the solution, and the mixture was filtered through Celite. The filtrate was washed with water and the organic layer was dried over Na_2SO_4 and evaporated to give Sb(III)PBr. The crude was redissolved in 50 mL of CH_3OH was added 0.3 mL of aq. H_2O_2 (30%). The mixture was stirred for 12 h at room temperature and the solvent then evaporated. At this point the counterion exchange was performed from Br^-/OH^- to PF_6^-. The crude was converted to a PF_6 salt by dissolving it in 10 mL of CH_3OH and adding NH_4PF_6 (660 mg, 4.05 mmol). The product was precipitated by the addition of water and collected by filtration and air dried. The crude was subjected to silica gel column chromatography, eluted with CH_2Cl_2 to remove unreacted free-base porphyrin H_2P, then polarity increased to CH_2Cl_2:EtOAc (= 95:5) to collect the product. Yield = 550 mg (73%). ESI-MS: *m/z* 781.1552 for $[M - PF_6]^+$, calcd 781.1558 for $C_{45}H_{32}N_4O_2Sb^+$. ^1H NMR ($CDCl_3$, 400 MHz): *δ, ppm* 9.49 (s, 8H), 8.49 (dd, 4H, *J* = 6.8 Hz), 8.31 (d, 4H, *J* = 7.6 Hz), 7.95 (m, 8H), 7.89 (m, 4H), 6.75 (bs, 2H), −2.21 (s, 3H), −3.31 (s, 1H). ^{31}P NMR ($CDCl_3$, 162 MHz): *δ, ppm* −146.32 (sept, 1P, *J* = 706 Hz).

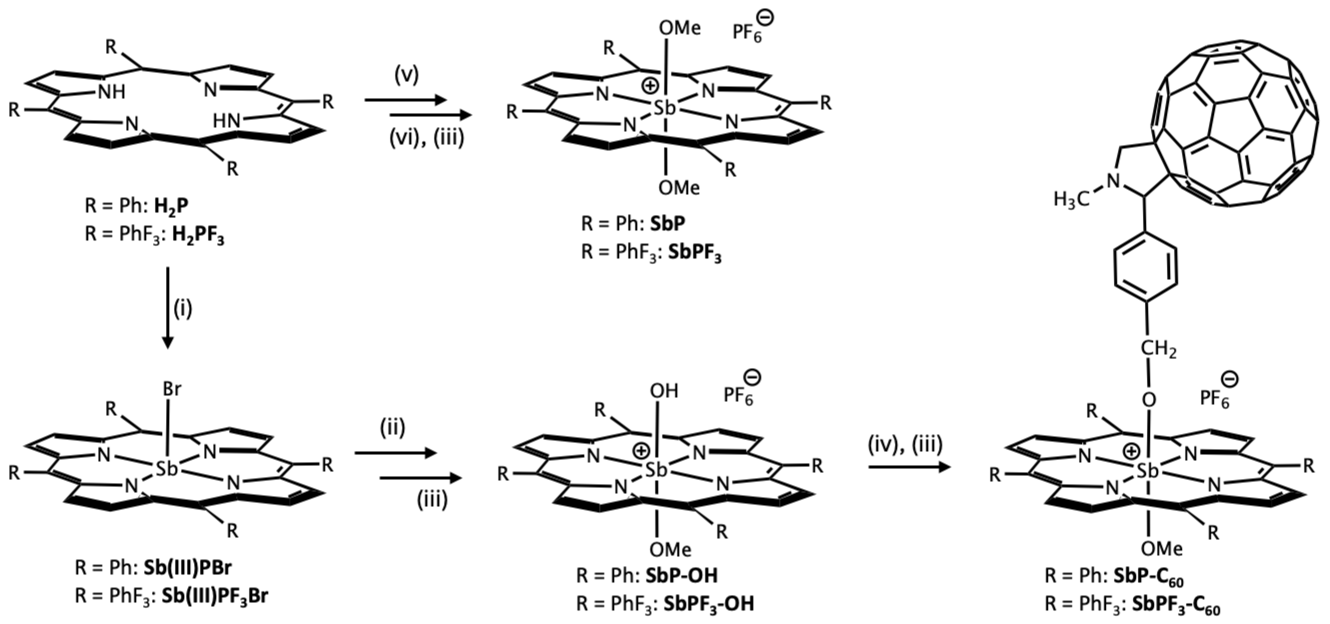

Scheme S2. Synthesis of antimony(V) porphyrin – C_{60} conjugates. *Reaction conditions*: (i) $SbBr_3$, dry CH_2Cl_2, 2,6-lutidine, reflux under N_2, 48-72 h, (ii) 30% H_2O_2, CH_3OH, stirring at room temperature, (iii) CH_3OH, NH_4PF_6, H_2O, (iv) C_{60}-Tol-Br, 18-crown-6, K_2CO_3, dry toluene, heat at 60°C under N_2, (v) $SbCl_5$, pyridine (vi) CH_3OH, $CHCl_3$, pyridine.

Synthesis of SbPF₃-OH. H_2PF_3 (220 mg, 0.27 mmol) and $SbBr_3$ (380 mg, 1.05 mmol) were heated under reflux for 72 h under N_2 in a solution containing 20 mL of dry CH_2Cl_2 and 0.45 mL of 2,6-Lutidine. After this time, the solvent was evaporated. It appears the Sb(III)PF_3Br is not readily soluble in CH_2Cl_2, therefore, celite filtration was skipped. The ESI-MS confirm the successful Sb(+3) ion insertion, showed *m/z* 948.9824 for $[M - Br]^+$, calcd 949.0216 for $C_{44}H_{16}F_{12}N_4Sb^+$. The crude Sb(III)$PF_3$Br was dissolved in 12 mL of CH_3OH was added 0.2 mL of aq. H_2O_2 (30%). The mixture was stirred for 12 h at room temperature and the solvent then evaporated. At this point the counterion exchange was performed from Br^-/OH^- to PF_6^-. The product was converted to a PF_6 salt by dissolving it in 10 mL of CH_3OH and adding NH_4PF_6 (220 mg, 1.35 mmol). The product was precipitated by the addition of water and collected by filtration and air dried. The crude was subjected to silica gel column chromatography, eluted with CH_2Cl_2 to remove unreacted free-base porphyrin H_2PF_3, then polarity increased to CH_2Cl_2:CH_3OH (= 95:3) to collect the pure product. Yield = 240 mg (80%). ESI-MS: *m/z* 997.0016 for $[M - PF_6]^+$, calcd 997.0427 for $C_{45}H_{20}F_{12}N_4O_2Sb^+$. ^1H NMR ($CDCl_3$, 400 MHz): *δ, ppm* 9.50 (s, 8H), 8.07 (t, 4H), 7.90 (t, 4H), −2.40 (s, 3H). ^{19}F NMR ($CDCl_3$, 375 MHz): *δ, ppm* −74.8 (d, 6F, *J* = 713 Hz), −131.7 (dt, 1F, *J* = 9.59 & 19.42 Hz), −133.8 (dt, 1F, *J* = 9.04 & 20.13 Hz), −156.9 (tt, 1F, *J* = 6.17 & 20.74 Hz). ^{31}P NMR ($CDCl_3$, 162 MHz): *δ, ppm* −146.12 (sept, 1P, *J* = 709 Hz).

Physical methods

NMR and mass spectroscopy. NMR spectra were recorded on a Bruker Advance 400 MHz NMR spectrometer using $CDCl_3$ as the solvent. ESI mass spectra were recorded on a Bruker MicroTOF-III mass spectrometer using acetonitrile as a solvent and direct injection from an UltiMate 3000 HPLC.

Absorption and emission spectroscopy. UV/Vis spectra were recorded with an Agilent Cary 100 UV/Vis spectrometer. The concentration of the samples used for these measurements ranged from 5×10^{-6} M (porphyrin Soret band) to 5×10^{-5} M (Q-bands) solutions. Steady-state fluorescence spectra were recorded using a Photon Technologies International Quanta Master 8075-11 spectrofluorometer, equipped with a 75 W xenon lamp, running with FelixGX software. The sample concentrations (1.50×10^{-5} M) were kept constant during the fluorescence studies. The excitation and emission slits of 1.0 or 1.5 nm were maintained.

Electrochemistry. Cyclic voltammetric experiments (in acetonitrile with 0.1 M tetrabutylammonium hexafluorophosphate (TBA.PF_6)) were performed on a BASi electrochemical analyzer (working electrode: Pt; auxiliary electrode: Pt wire; reference electrode: Ag wire). The Fc^+/Fc (Fc = ferrocene, $E_{1/2}(Fc^+/Fc)$ = 0.46 V *vs*. SCE in CH_2Cl_2, 0.1 M TBA.PF_6 under these experimental conditions) redox couple was used to calibrate the potentials.

DFT calculations. The conjugate structures were initially constructed on a local PC using the *GaussView 6* (GV6.0) software. DFT computations were performed on a supercomputer using the *Gaussian 16* software suite. Since the investigation includes the theoretical study of the excited state and charge transfer properties where the highest excitation is to the LUMO+1 the B3LYP method was chosen. The 6-311G(df,pd) split-valence polarized basis set was used to model hydrogen and the period 2 elements (C, N, O, and F) in the compounds. Since antimony is a period 5 element, the relativistic effects of the core electrons was modeled using effective core potentials (ECPs). The Stuttgart/Dresden ECPs in combination with the *triple-zeta* polarized basis set (def2TZVPP) was chosen to model antimony for this study. Thus, the B3LYP method was coupled with a GenECP basis to form the B3LYP/GenECP model chemistry which was used to optimize the geometry of all the structures herein to a stationary point on the Born-Oppenheimer surface and calculate the first ten excited singlet states of all chemical species in the current study. The optimization process was followed by a frequency calculation, utilizing the same model chemistry used in the optimization, to ensure that the stationary point to which the molecules were optimized was a minimum. All the structures were

optimized *sans* symmetry constraints *in vacuo* as +1 charged cations and closed-shell singlets. The self-consistent field (SCF) convergence constraints and the DFT grid utilized in the calculation were the G16 default values, “Tight” and “UltraFine” respectively. The optimization of the geometrical parameters of each of the chemical species in the study was continued until the maximum force, RMS force, maximum displacement, and RMS displacement reached or was less than, the default *Gaussian 16* minima and the predicted energy change upon a successive optimization cycle of the geometrical parameters was in the range of $-5x10^{-9}$ A.U.

Femtosecond laser flash photolysis. Femtosecond transient studies were performed using an Ultrafast Femtosecond Laser Source (Astrella) by Coherent, which incorporates a diode, mode-locked Ti: Sapphire laser (Vitara), and diode-pumped intracavity doubled Nd: YLF laser (Revolution) to generate a fundamental compressed laser of 800 nm and power output of 5.24 W. A Helios transient absorption spectrometer coupled with a femtosecond harmonics generator, both provided by Ultrafast Systems LLC, will be used for optical detection. The source for the pump pulse is derived from the fundamental output of Astrella (compressed output 5.24 W, pulse width 100 fs, 800 nm at a repetition rate of 1 kHz) by introducing 95% of the beam into the OPA while the other 5% is sent to the delay line and white light generating crystal. The beam sent through the OPA is termed the pump beam, as it is used to excite the sample. The beam sent through the delay line and crystal is termed the probe beam as it shows what spectral changes occur in the sample with time. The OPA takes the 800 nm fundamental and converts it into a specific wavelength in the 400 – 2200 nm range, which allows the excitation wavelength to be selected. Kinetic traces at appropriate wavelengths were assembled from the time-resolved spectral data. Data analysis was performed using Surface Xplorer software. All measurements were conducted in degassed solutions at 298 K.

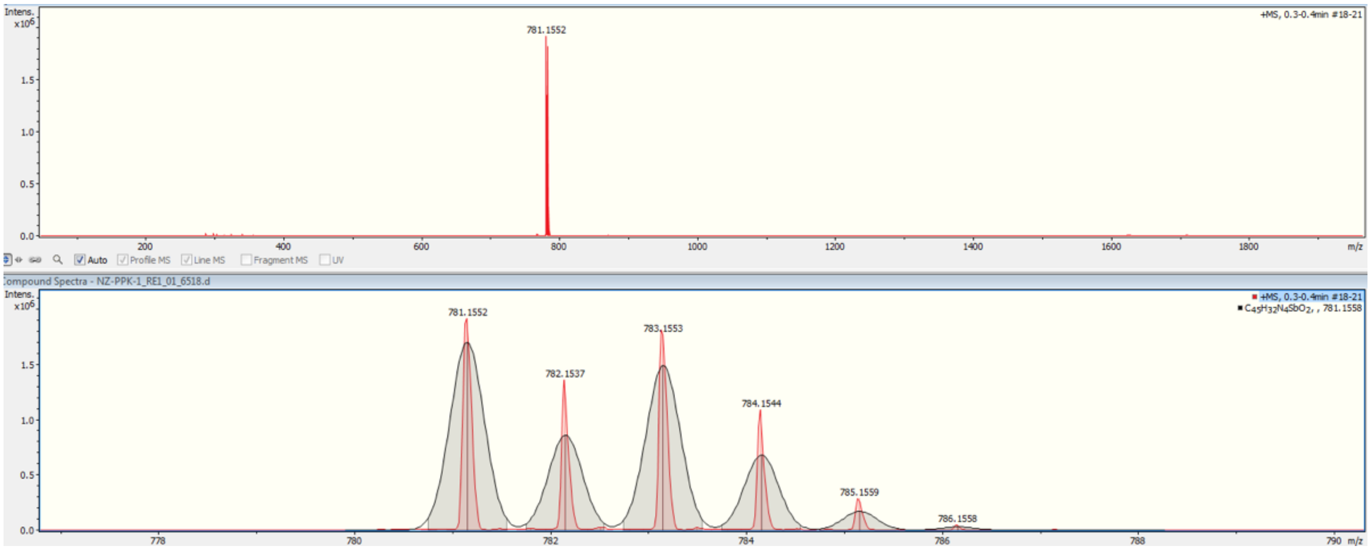

Figure S1. HS-ESI mass spectrum of the SbP-OH. Simulations are indicated in black.

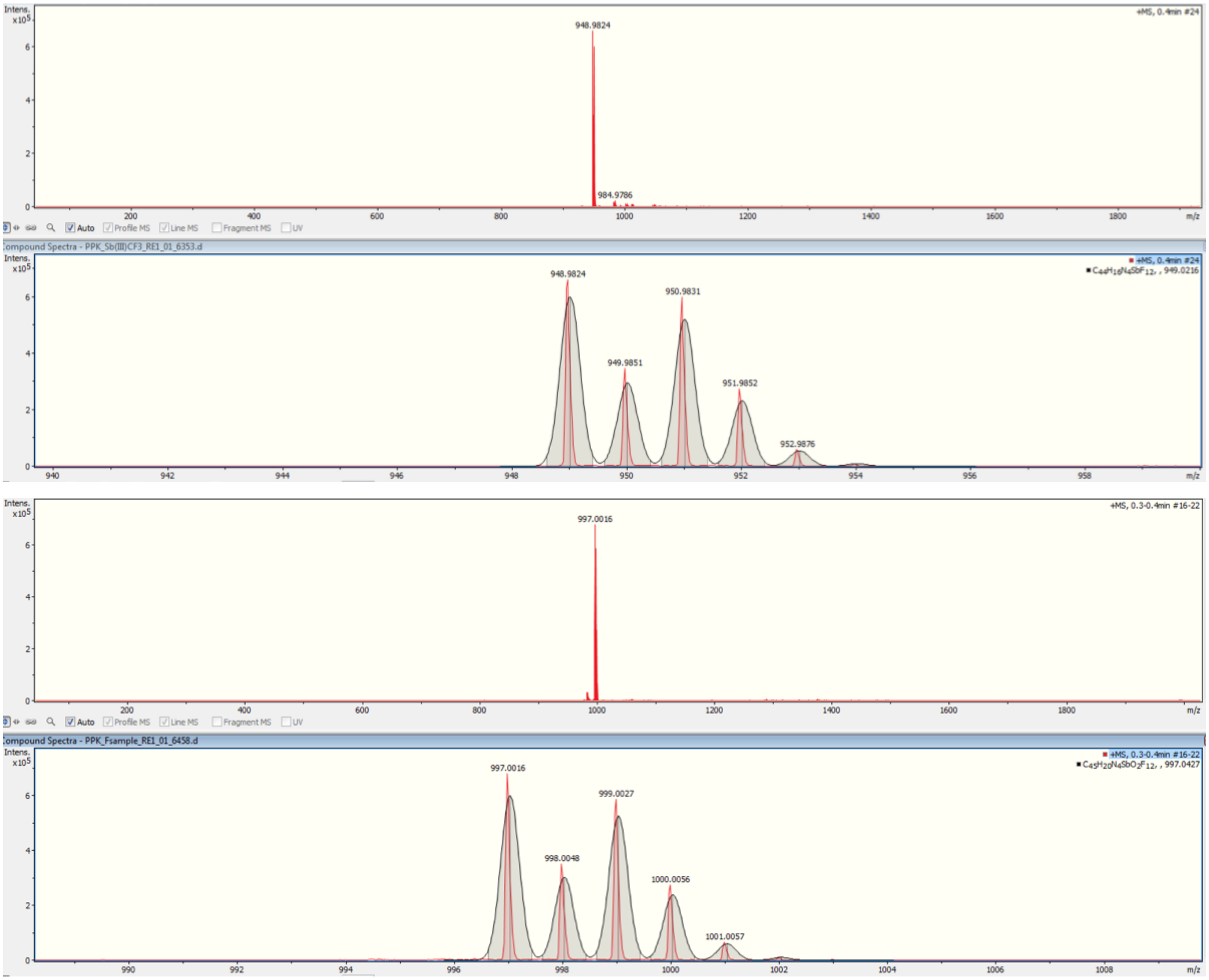

Figure S2. HS-ESI mass spectra of the Sb(III)PF_3Br (top) and SbPF_3-OH (bottom). Simulations are indicated in black.

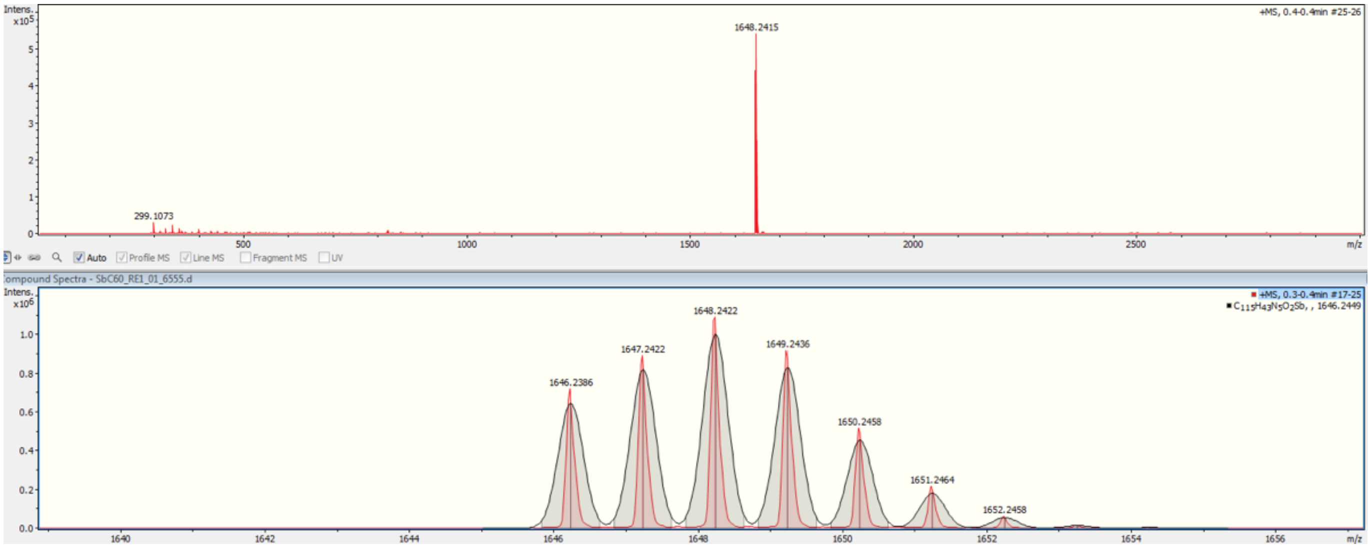

Figure S3. HS-ESI mass spectrum of the SbP-C_{60}. Simulations are indicated in black.

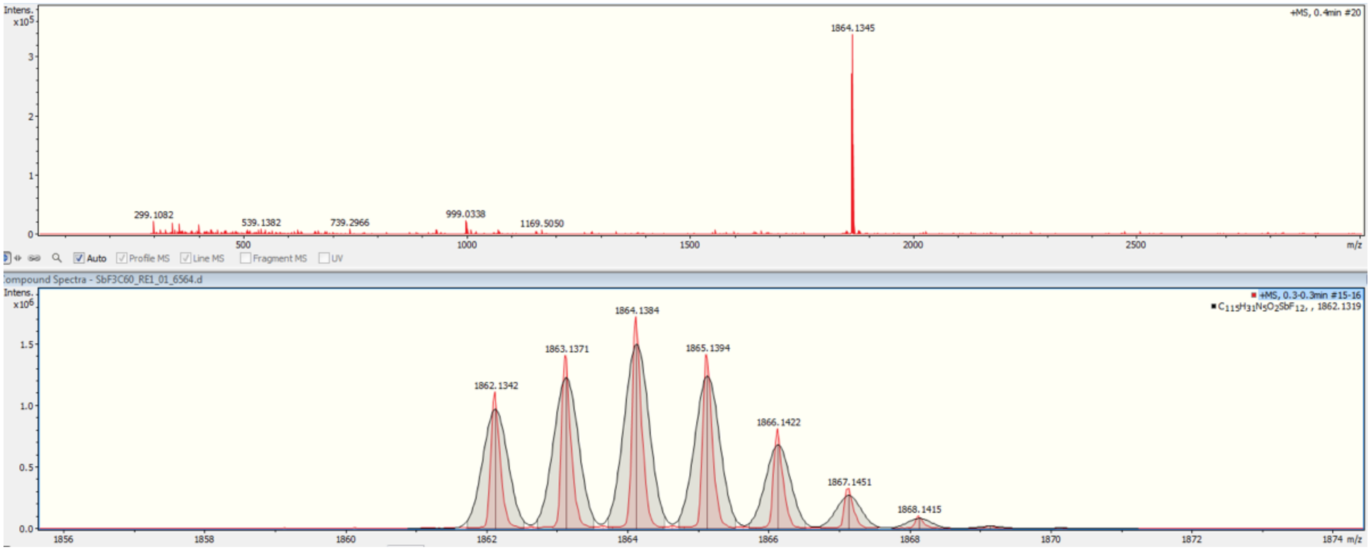

Figure S4. HS-ESI mass spectrum of the $SbPF_3$-C_{60}. Simulations are indicated in black.

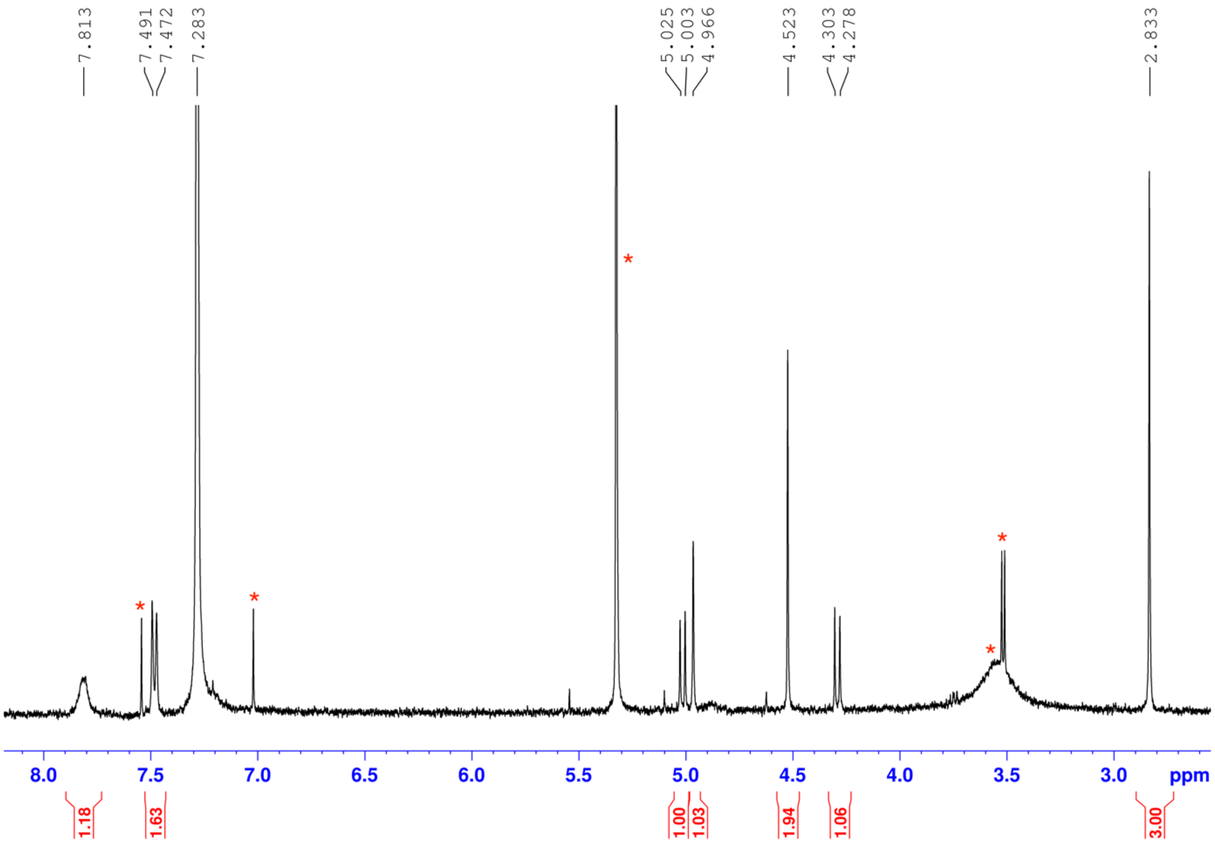

Figure S5. ^{1}H (400 MHz) NMR spectrum of the C_{60}-Tol-Br in $CDCl_3$. * Indicates solvent impurities.

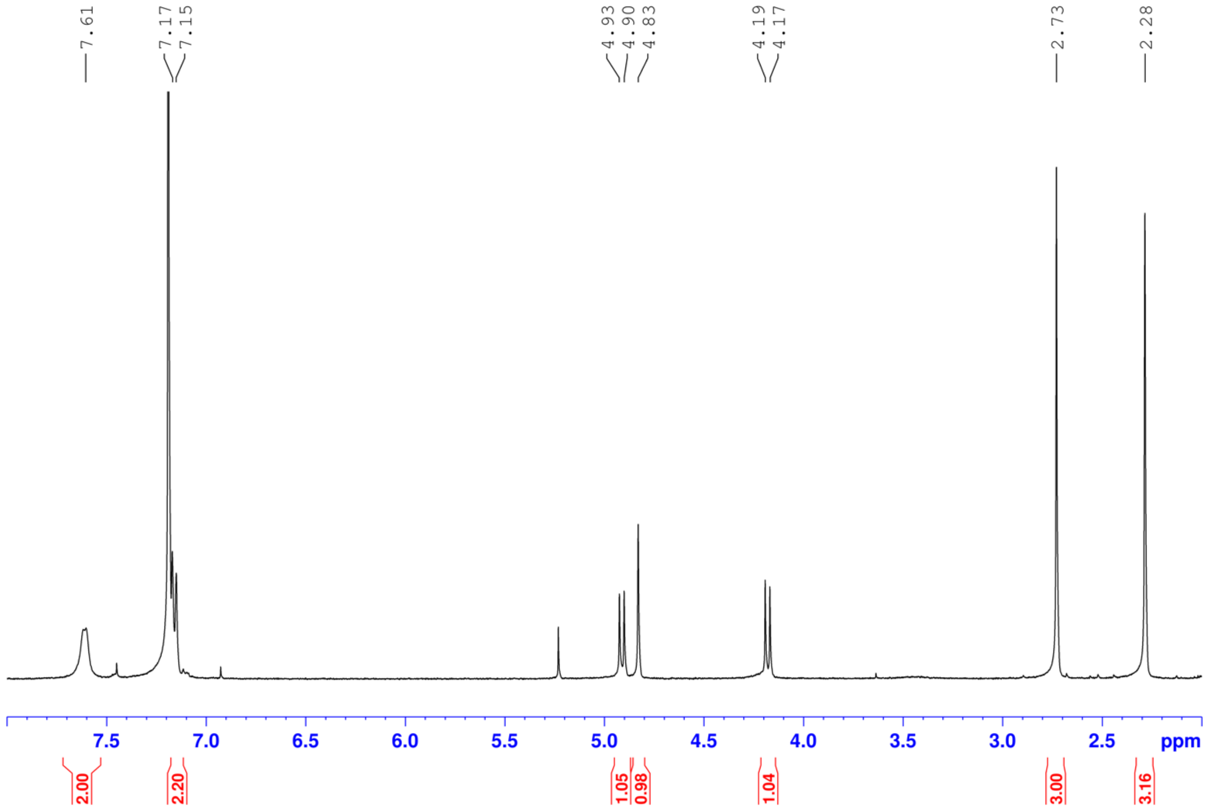

Figure S6. ^{1}H (400 MHz) NMR spectrum of the C_{60}-Tol in $CDCl_3$.

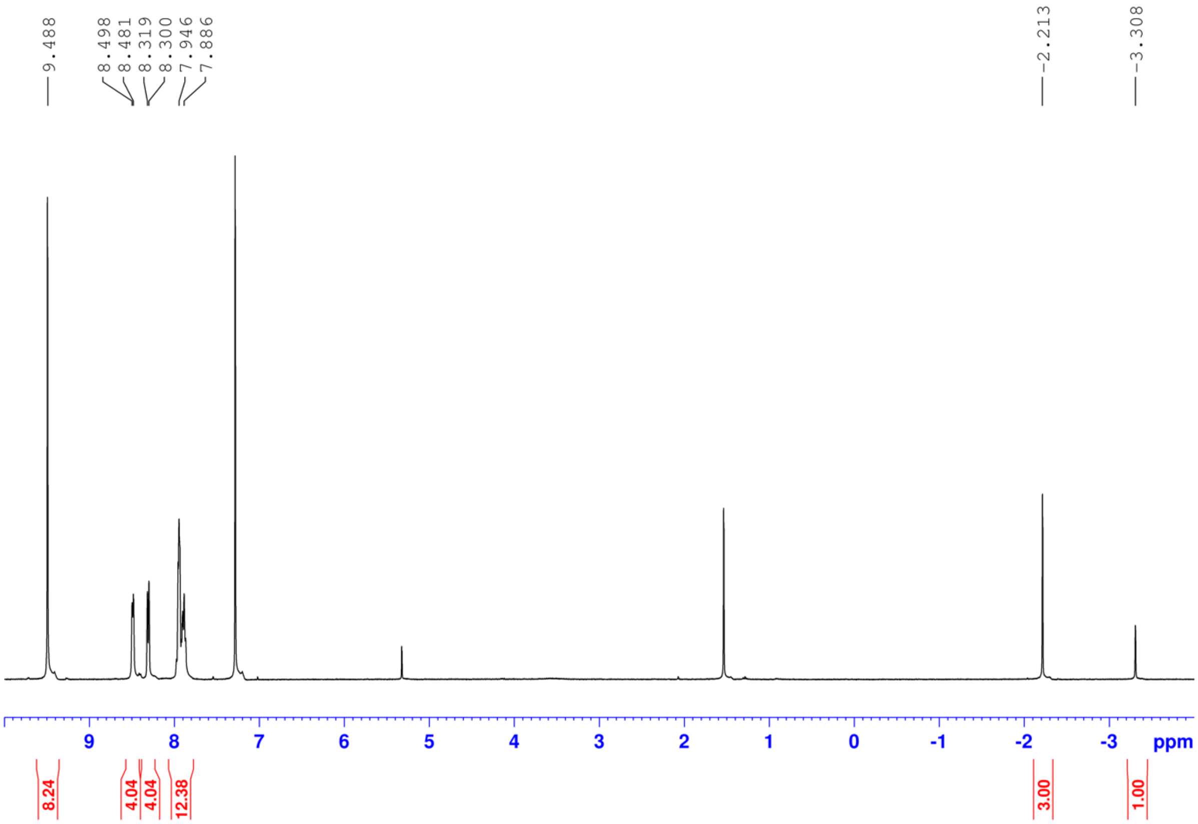

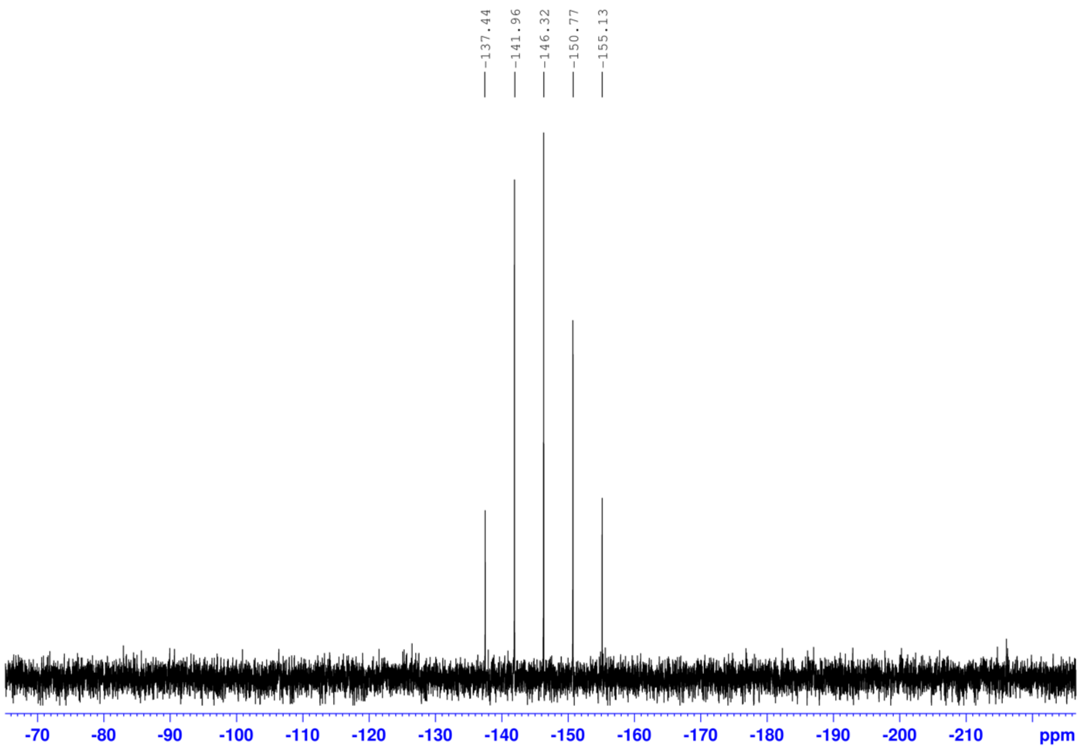

Figure S7. ^{1}H (400 MHz) and ^{31}P (162 MHz) NMR spectra of the SbP-OH in $CDCl_3$.

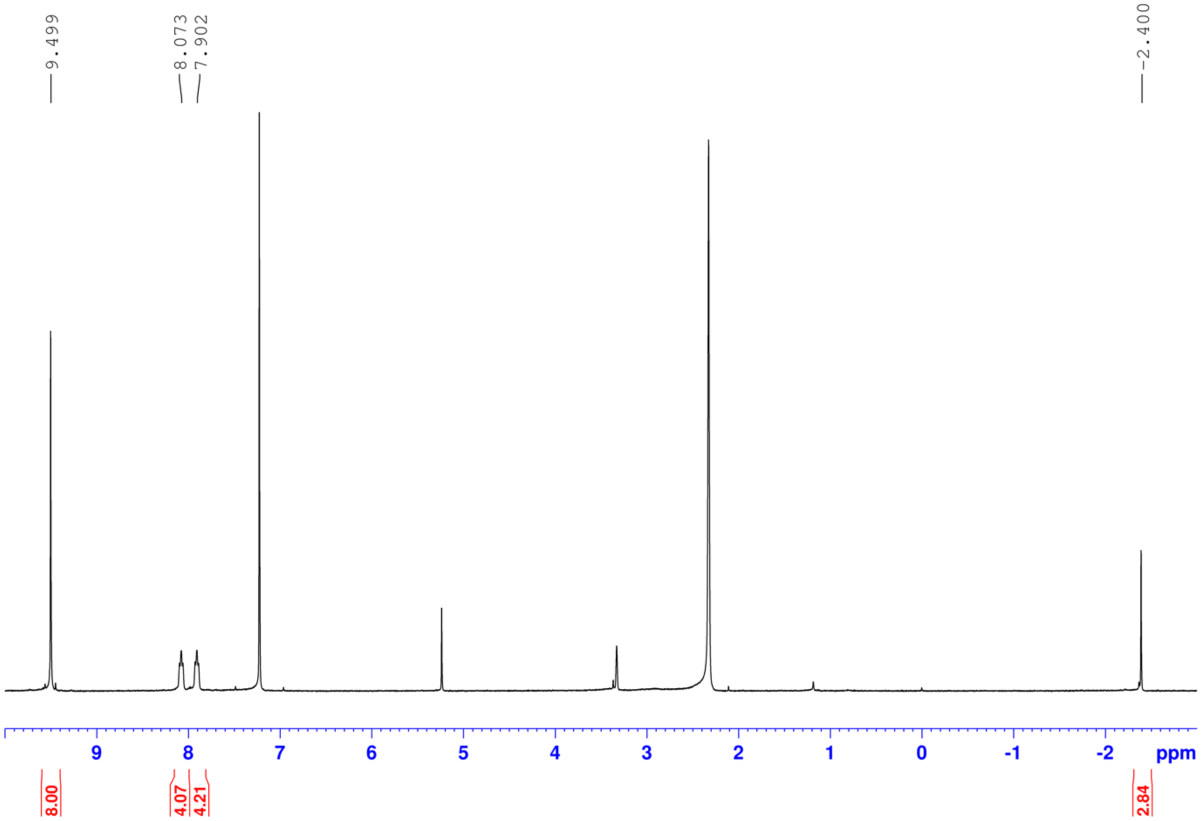
9.499
8.073
7.902
-2.400
9
8
7
6
5
4
3
2
1
0
-1
-2
ppm
8.00
4.07
4.21
2.84

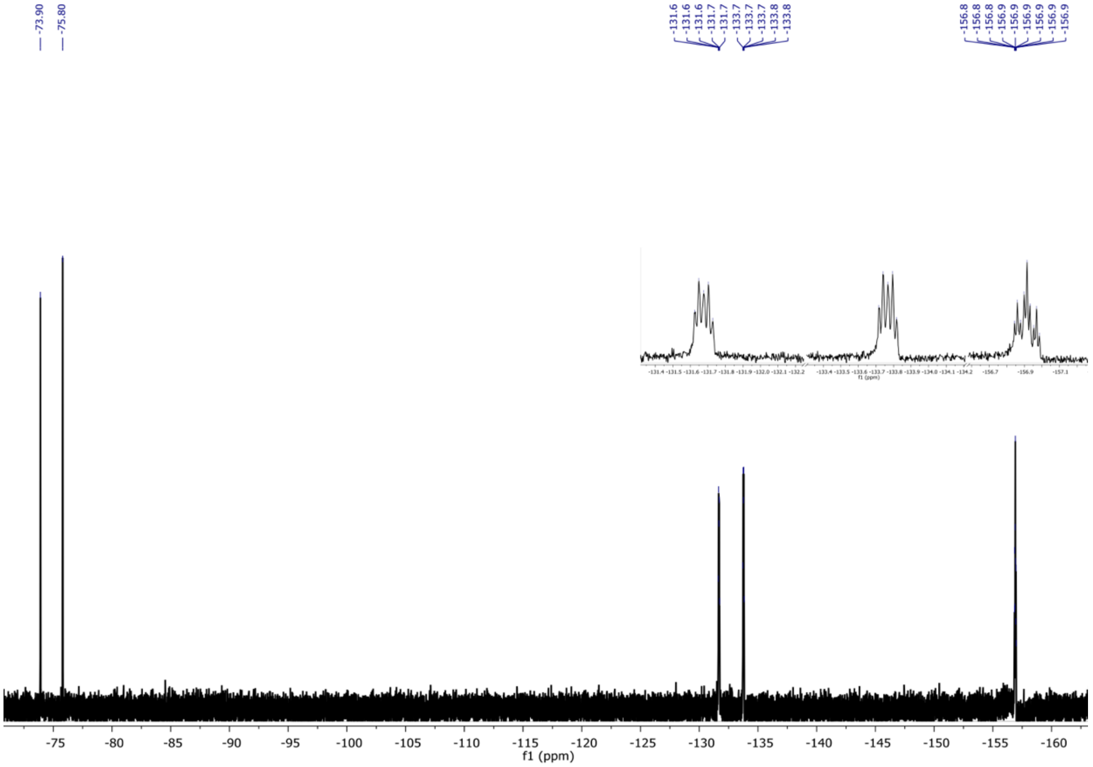
-73.90
-75.80
-131.6
-131.6
-131.7
-131.7
-133.7
-133.7
-133.7
-133.8
-133.8
-156.8
-156.8
-156.8
-156.9
-156.9
-156.9
-156.9
-156.9
-75
-80
-85
-90
-95
-100
-105
-110
-115
-120
-125
-130
-135
-140
-145
-150
-155
-160
f1 (ppm)

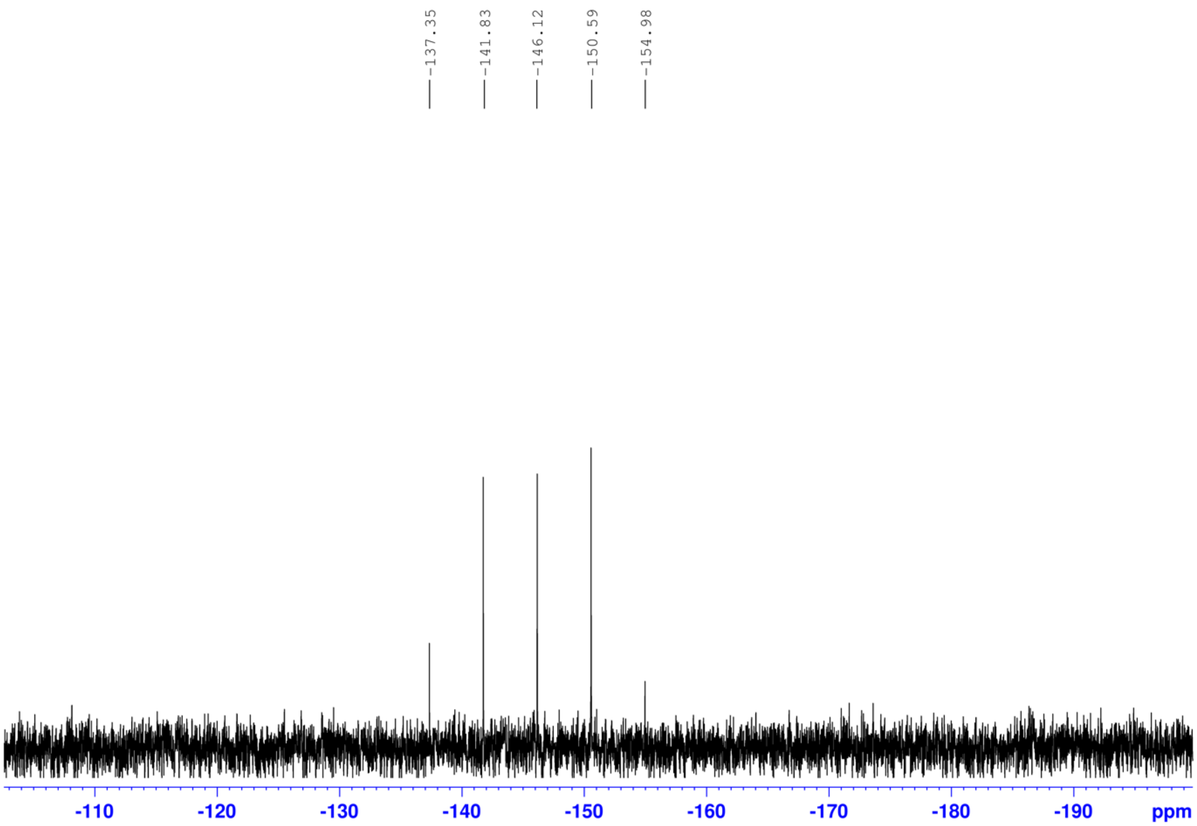

Figure S8. ^{1}H (400 MHz), ^{19}F (375 MHz), and ^{31}P (162 MHz) NMR spectra of the $SbPF_3$-OH in $CDCl_3$.

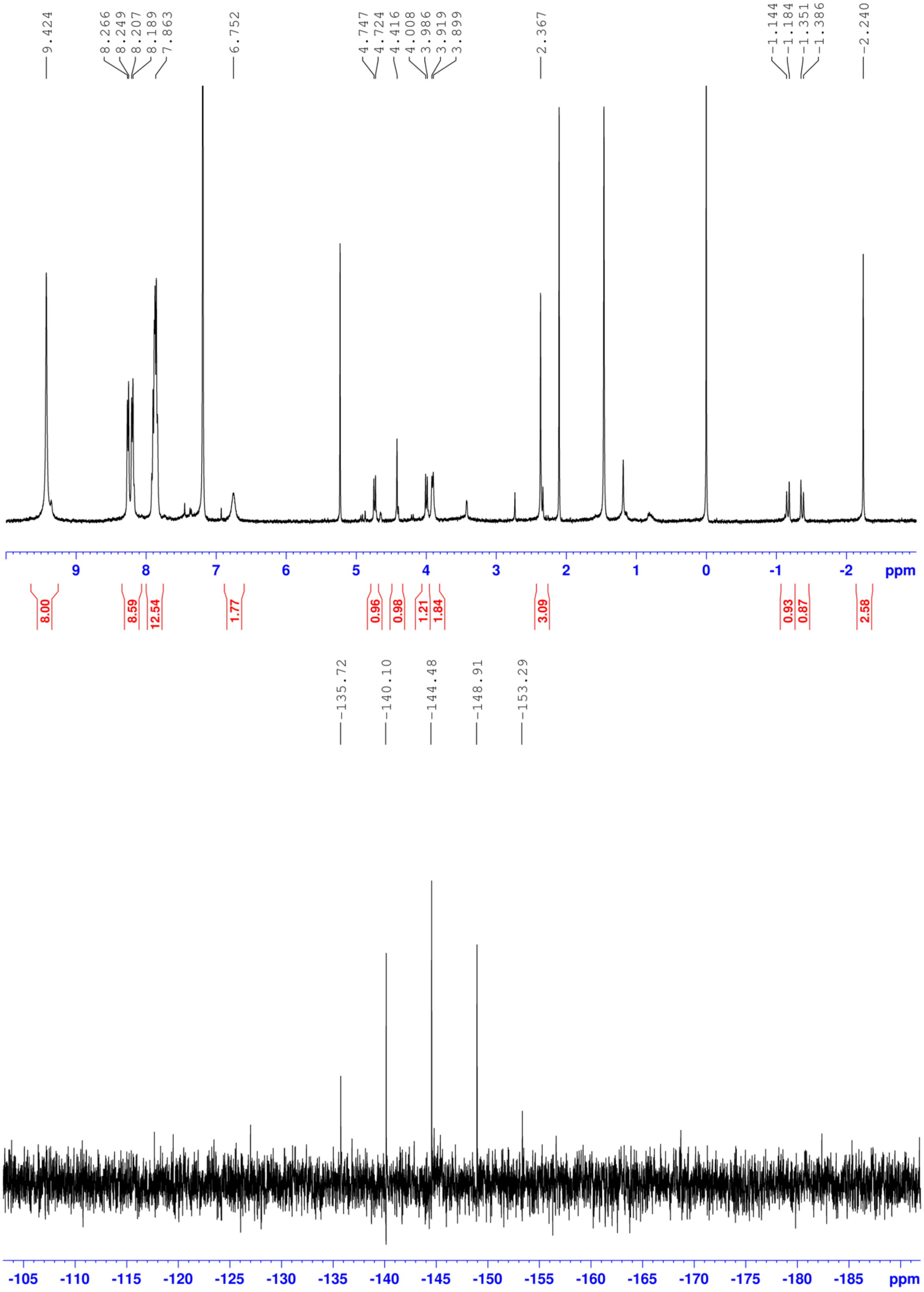

Figure S9. ^{1}H (400 MHz) and ^{31}P (162 MHz) NMR spectra of the SbP-C_{60} in $CDCl_3$.

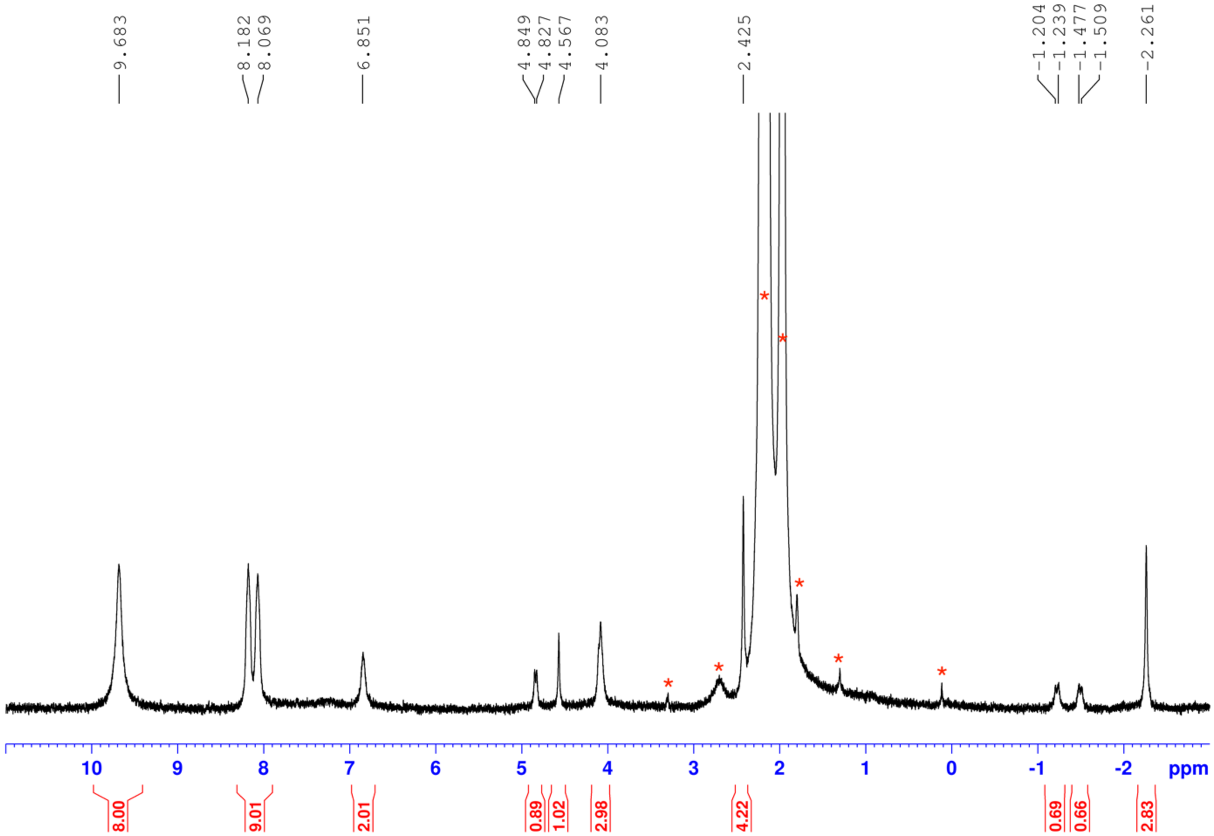

9.683
8.182
8.069
6.851
4.849
4.827
4.567
4.083
2.425
-1.204
-1.239
-1.477
-1.509
-2.261
10
9
8
7
6
5
4
3
2
1
0
-1
-2
ppm
8.00
9.01
2.01
0.89
1.02
2.98
4.22
0.69
0.66
2.83

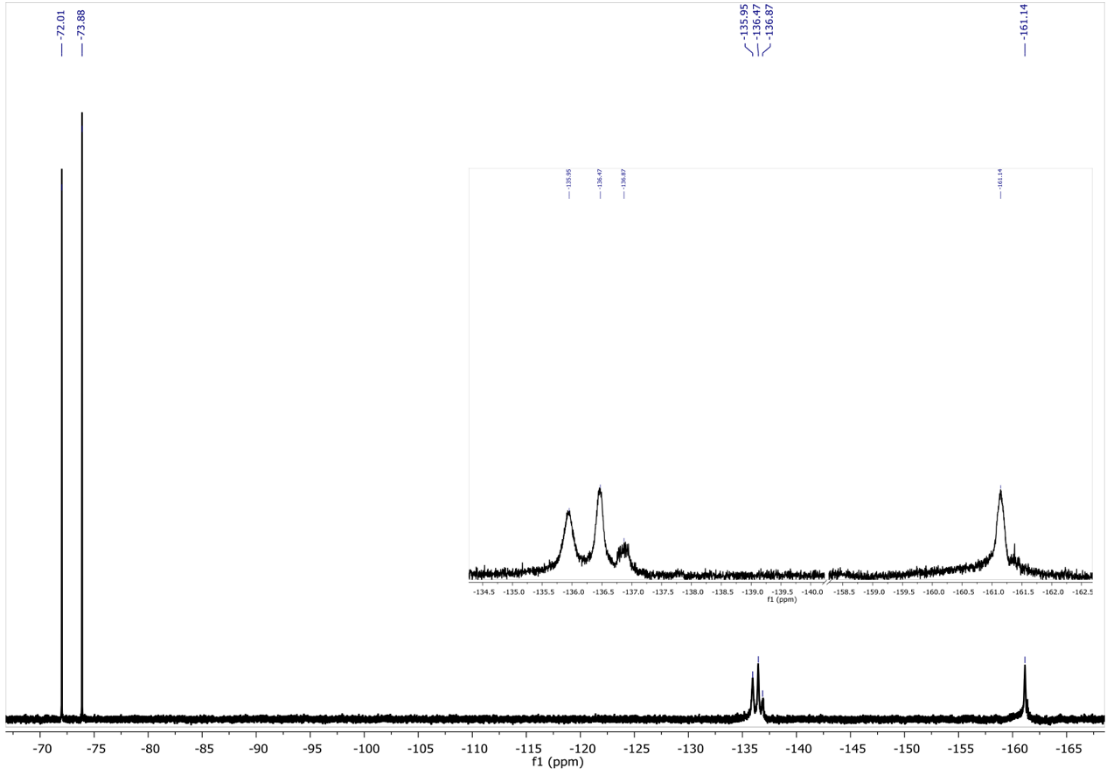

-72.01
-73.88
-135.95
-136.47
-136.87
-161.14
f1 (ppm)

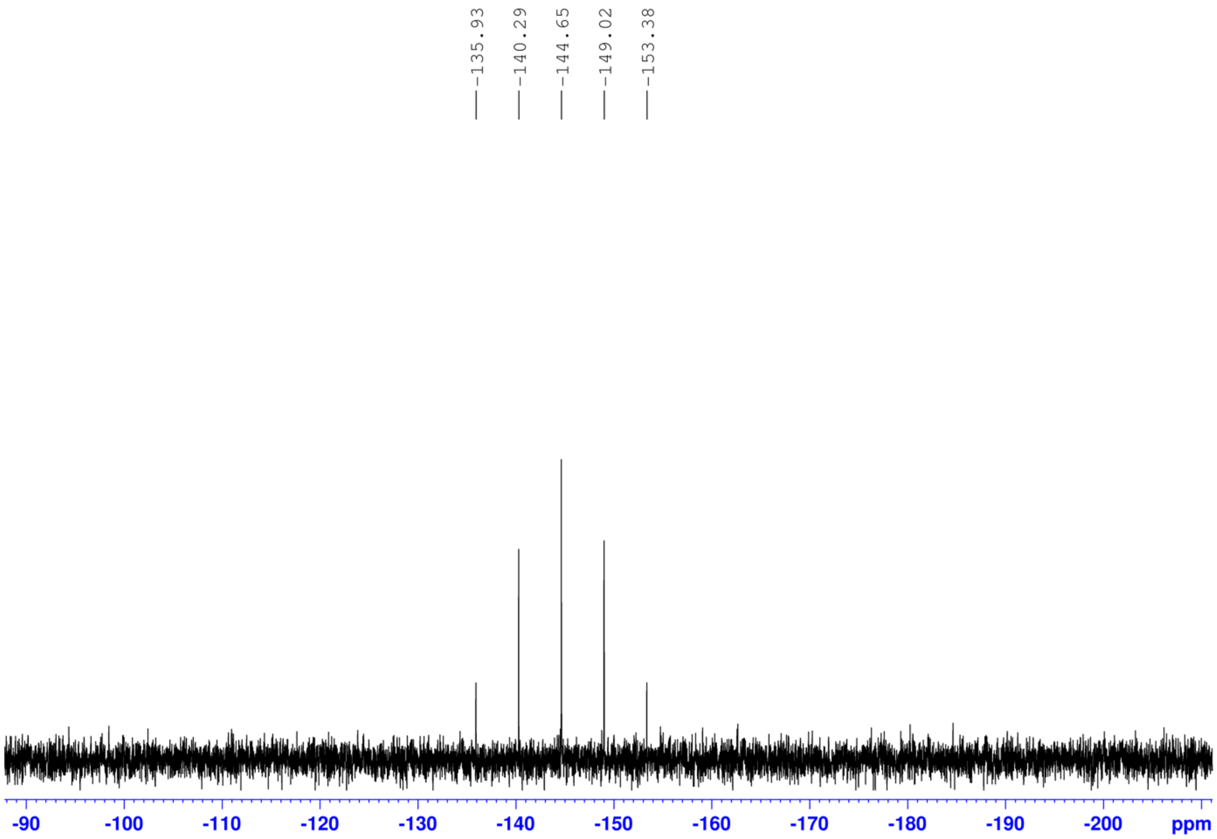

Figure S10. ^{1}H (400 MHz), ^{19}F (375 MHz), and ^{31}P (162 MHz) NMR spectra of the $SbPF_3$-C_{60} in CD_3CN.
* Indicates solvent peaks.

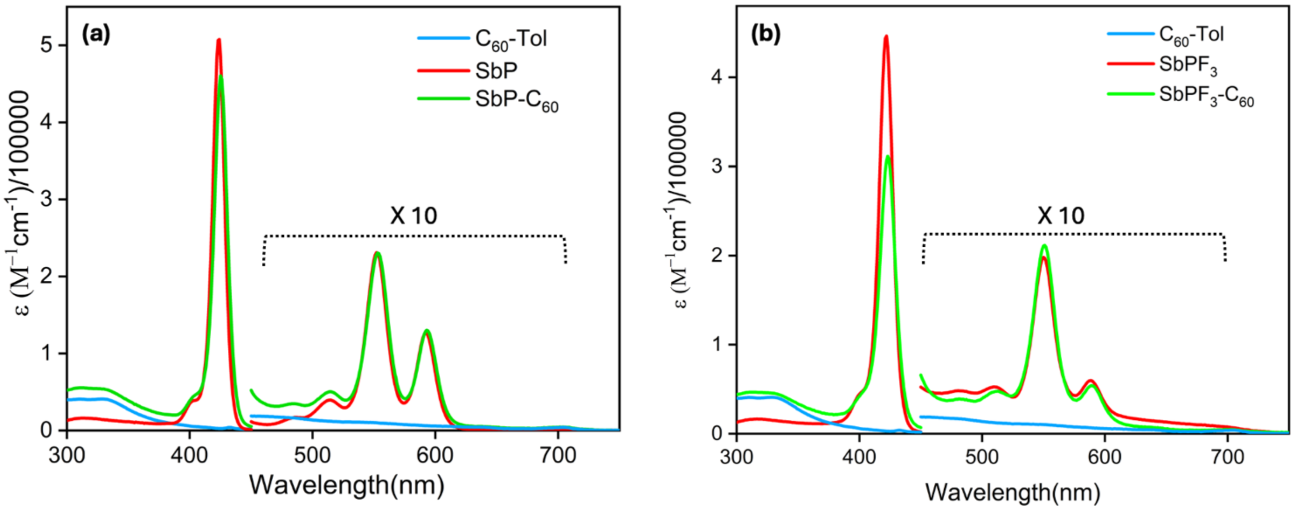

Figure S11. Electronic absorption spectra of (a) SbP-C_{60} and its reference compounds SbP and C_{60}-Tol; (b) $SbPF_3$-C_{60} and its reference compounds $SbPF_3$ and C_{60}-Tol in toluene.

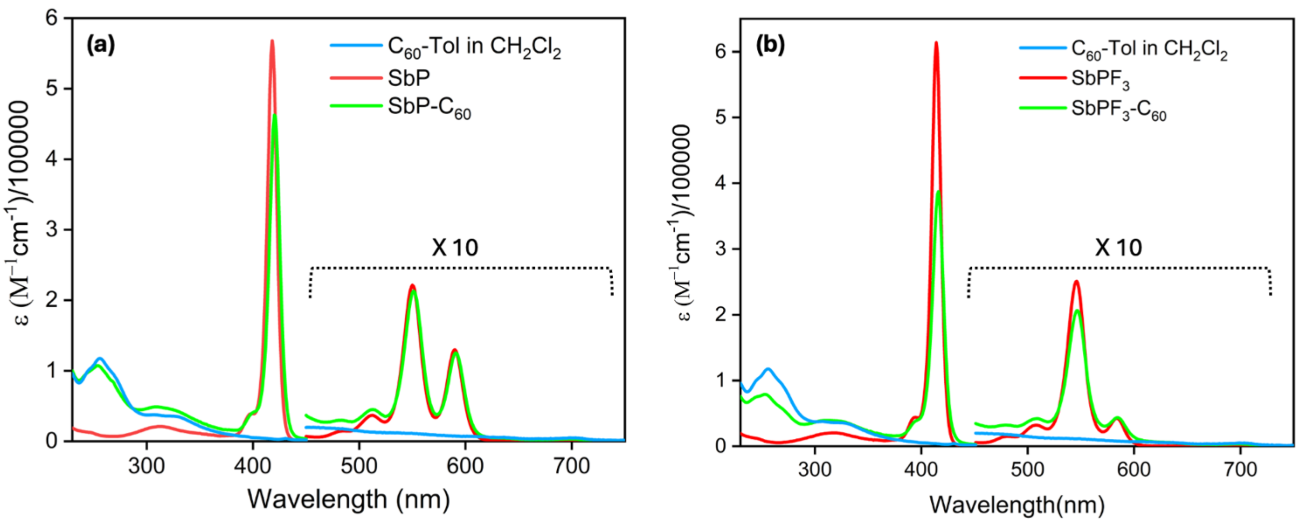

Figure S12. Electronic absorption spectra of (a) SbP-C_{60} and its reference compounds SbP and C_{60}-Tol; (b) $SbPF_3$-C_{60} and its reference compounds $SbPF_3$ and C_{60}-Tol in CH_3CN. For solubility reasons C_{60}-Tol is reported in CH_2Cl_2.

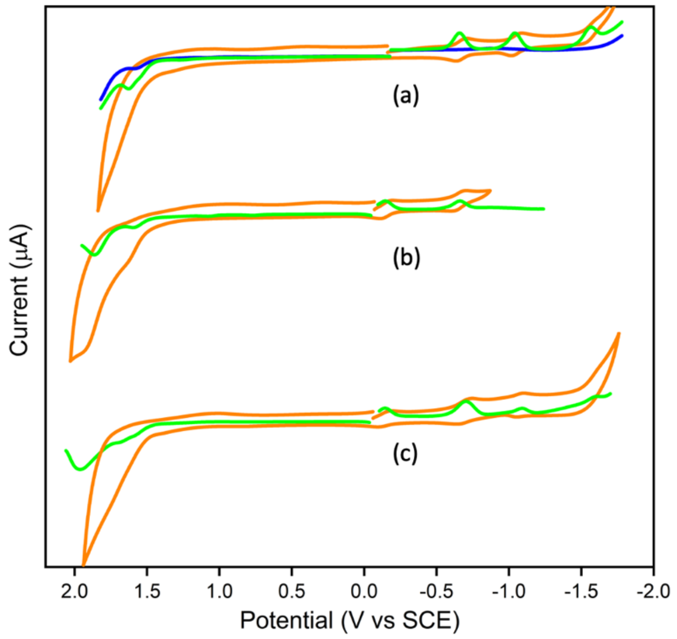

Figure S13. Cyclic (orange) and differential (green) voltammograms of (a) C_{60}-Tol, (b) $SbPF_3$, and (c) $SbPF_3$-C_{60} with 0.1 M TBA.PF_6 in CH_2Cl_2. For comparison purpose voltammogram (blue) of CH_2Cl_2 also displayed.

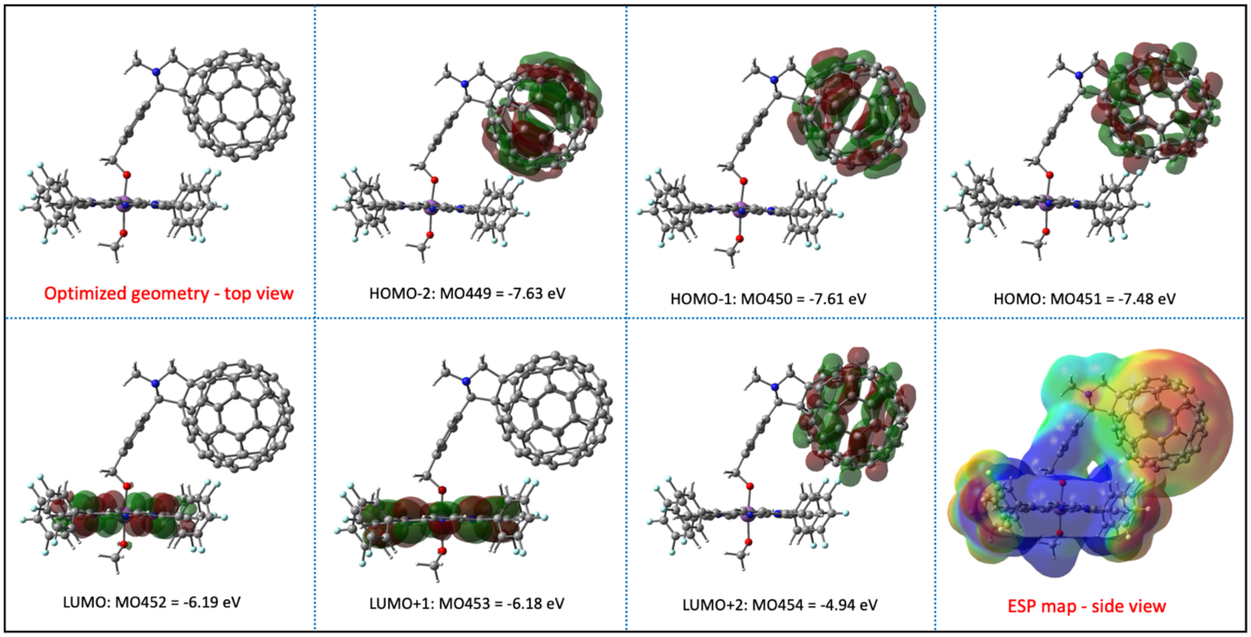

Figure S14. Structurally optimized $SbPF_3$-C_{60} conjugate and their DFT calculated frontier molecular orbitals, energies and electrostatic potential maps. Blue = electron acceptor region. Red = electron donor region.

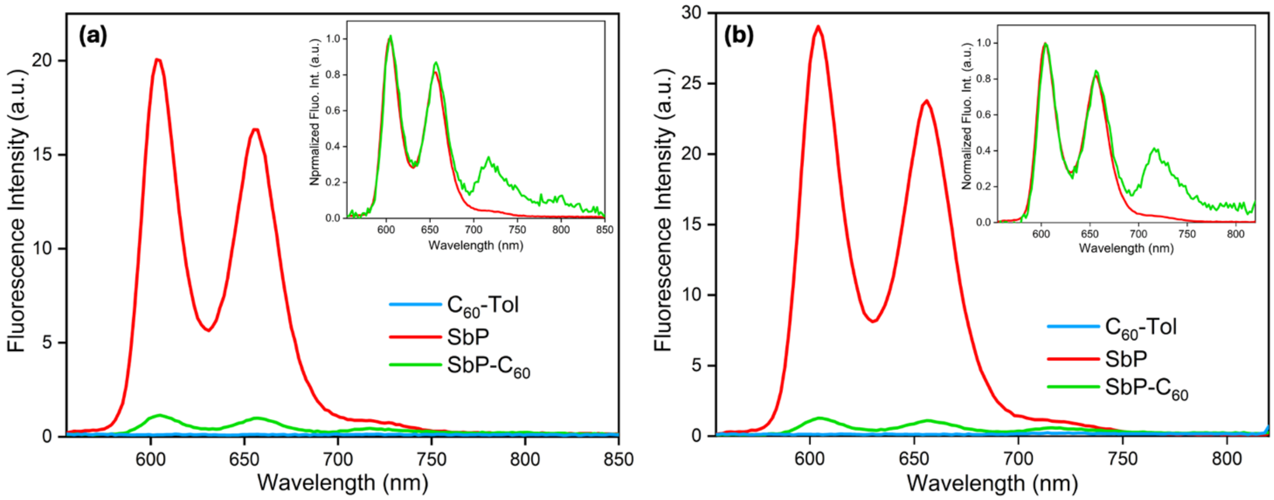

Figure S15. Fluorescence spectra of SbP-C_{60}, SbP, and C_{60}-Tol in toluene at excitation (a) 545 nm, and (b) 410 nm. Inset shows the normalized fluorescence spectra of SbP-C_{60} and SbP.

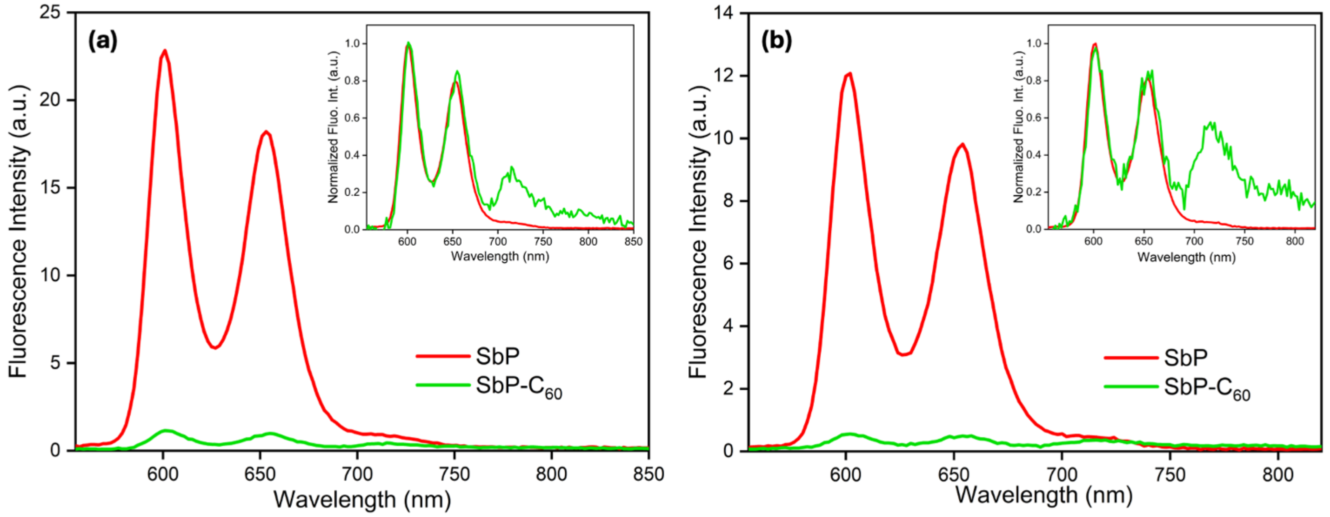

Figure S16. Fluorescence spectra of SbP-C_{60}, SbP, and C_{60}-Tol in CH_3CN at excitation (a) 545 nm, and (b) 410 nm. Inset shows the normalized fluorescence spectra of SbP-C_{60} and SbP.

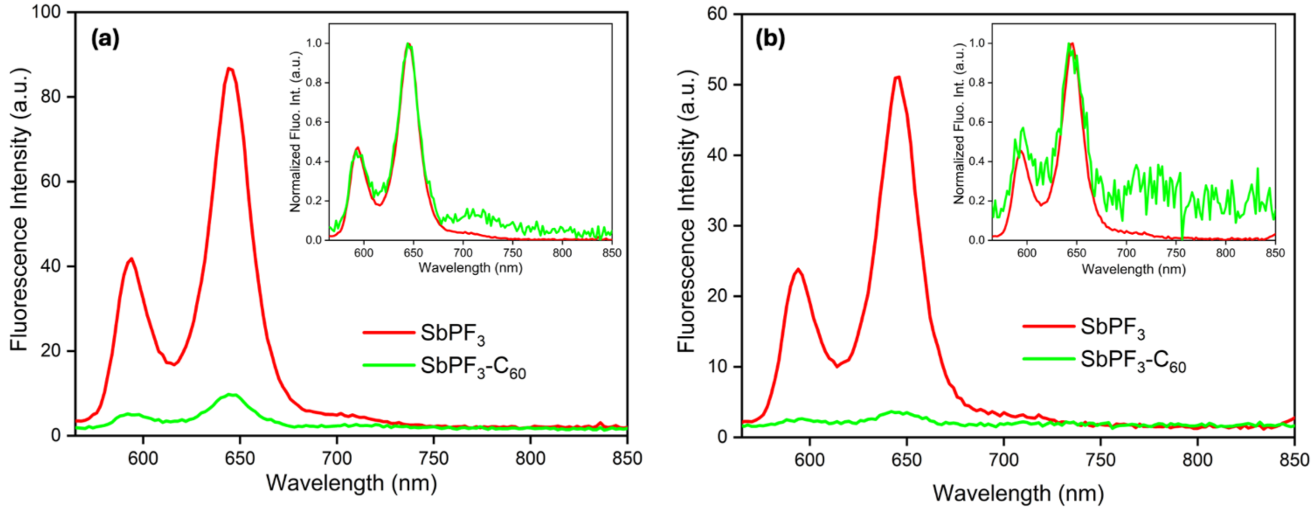

Figure S17. Fluorescence spectra of $SbPF_3$-C_{60}, and $SbPF_3$ in CH_3CN at excitation (a) 555 nm, and (b) 420 nm. Inset shows the normalized fluorescence spectra of $SbPF_3$-C_{60} and $SbPF_3$.

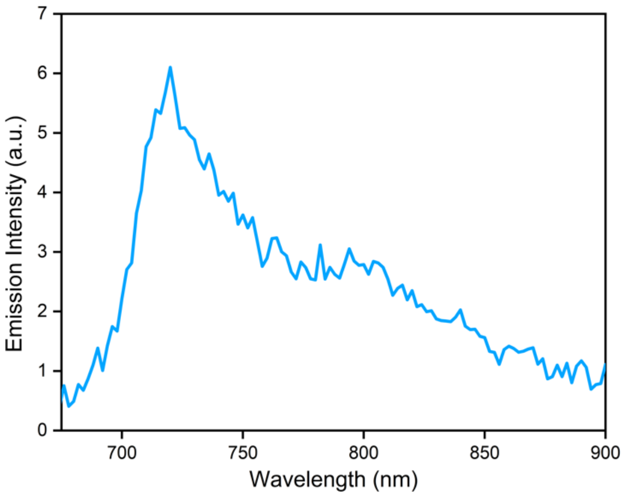

Figure S18. Fluorescence spectrum of C_{60}-Tol in o-DCB at excitation 330 nm.

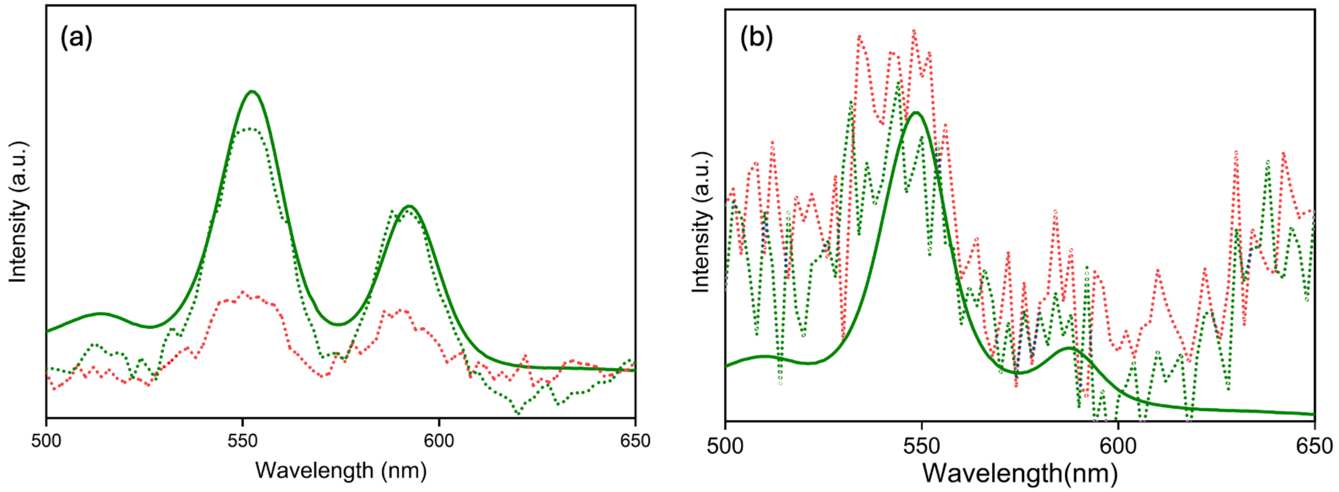

Figure S19. (a) Normalized absorption (solid green) and excitation (dotted green) spectra of SbP-C_{60}, and absorption spectra of SbP (b) Normalized absorption (solid green) and excitation (dotted green) spectra of $SbPF_3$-C_{60}, and absorption spectra of $SbPF_3$ in CH_2Cl_2. All the sample concentrations maintained to be ~ 1.50 × 10^{-5} M. *Note that the relative intensities between conjugates and their reference porphyrin are maintained during the normalization process.*

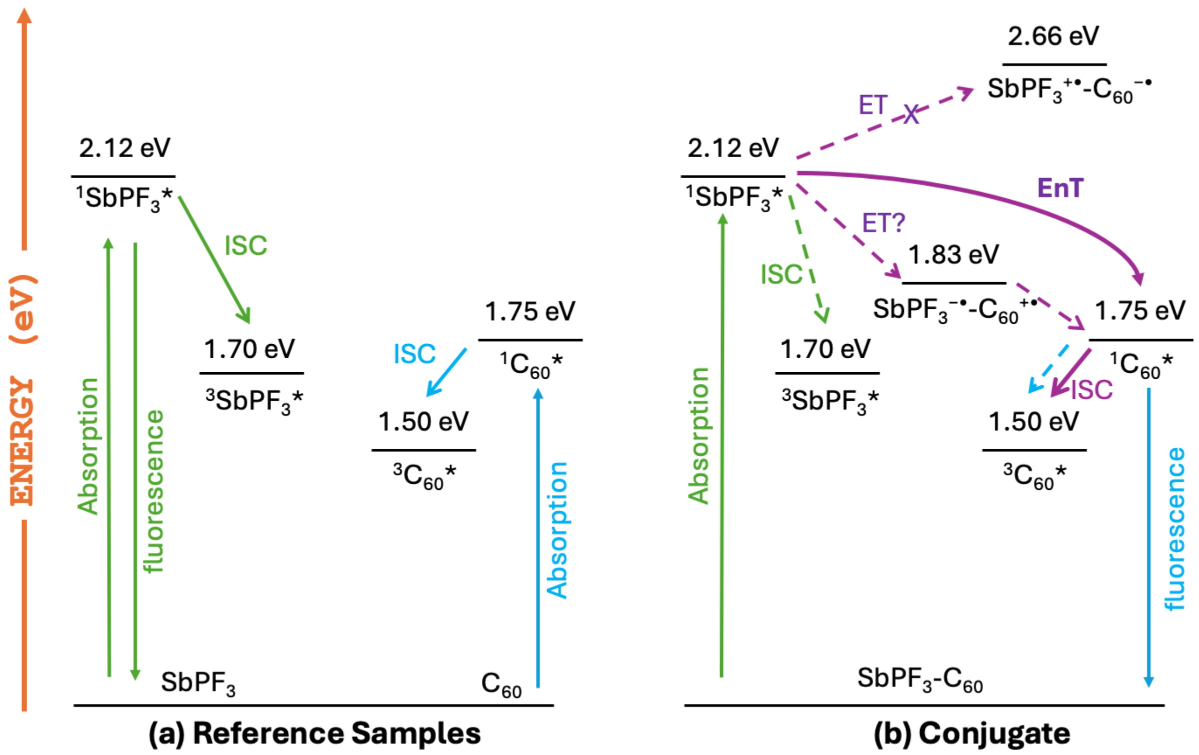

Figure S20. Energy level diagram of (a) reference compounds $SbPF_3$ and C_{60}-Tol, and (b) conjugate $SbPF_3$-C_{60} in CH_2Cl_2. Solid and dashed arrows represent directly detected and undetected processes, respectively.

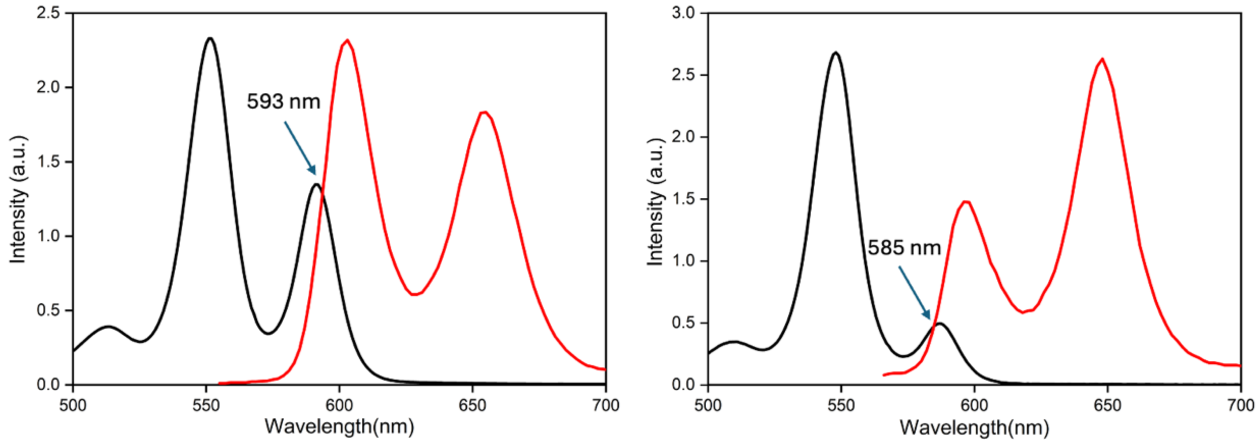

Figure S21. Overlap of absorption (black) and fluorescence (red) spectra of (left) SbP and (right) $SbPF_3$ in CH_2Cl_2.

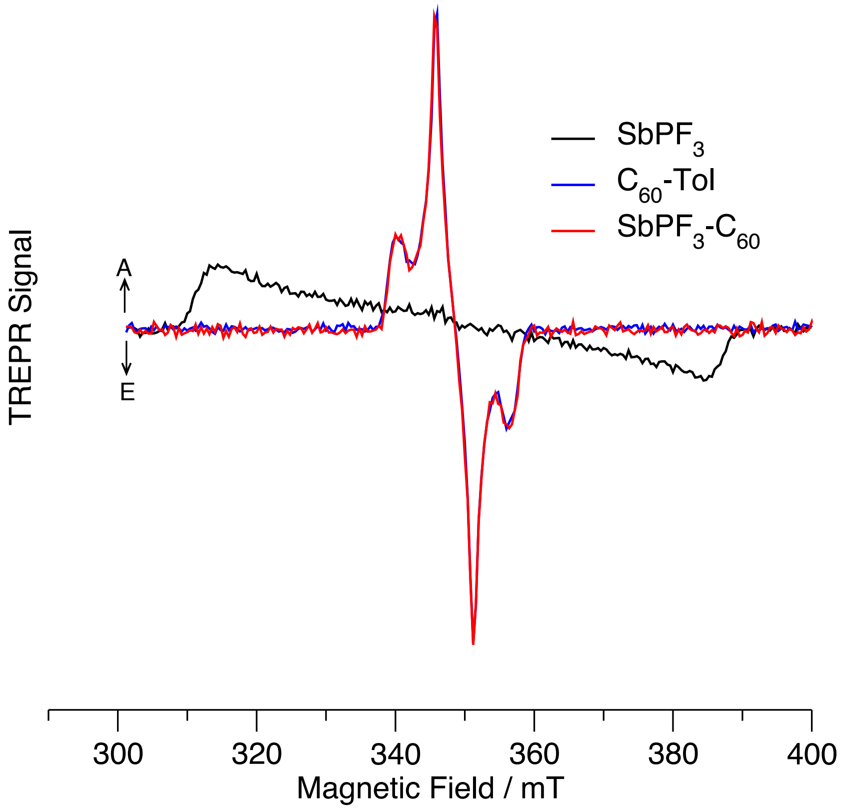

Figure S22. Spin polarized transient EPR spectra of $SbPF_3$, C_{60}, and $SbPF_3$-C_{60} measured at 80 K in MeTHF. The spectra are the average signal intensity a 700 ns wide time window centered at 1.75 μs after the laser flash.

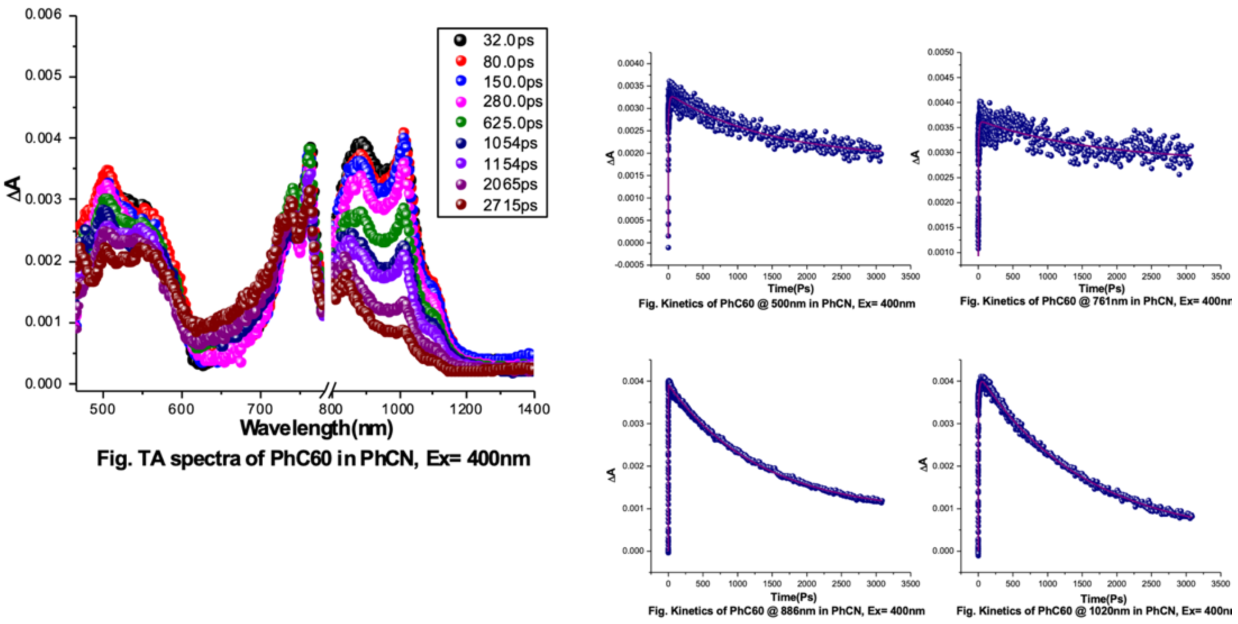

Figure S23. Nanosecond transient absorption spectra at indicated delay times (λ_{ex} = 400 nm) of C_{60}-Tol in PhCN. The inset shows the decay profile of the mentioned triplet peak.

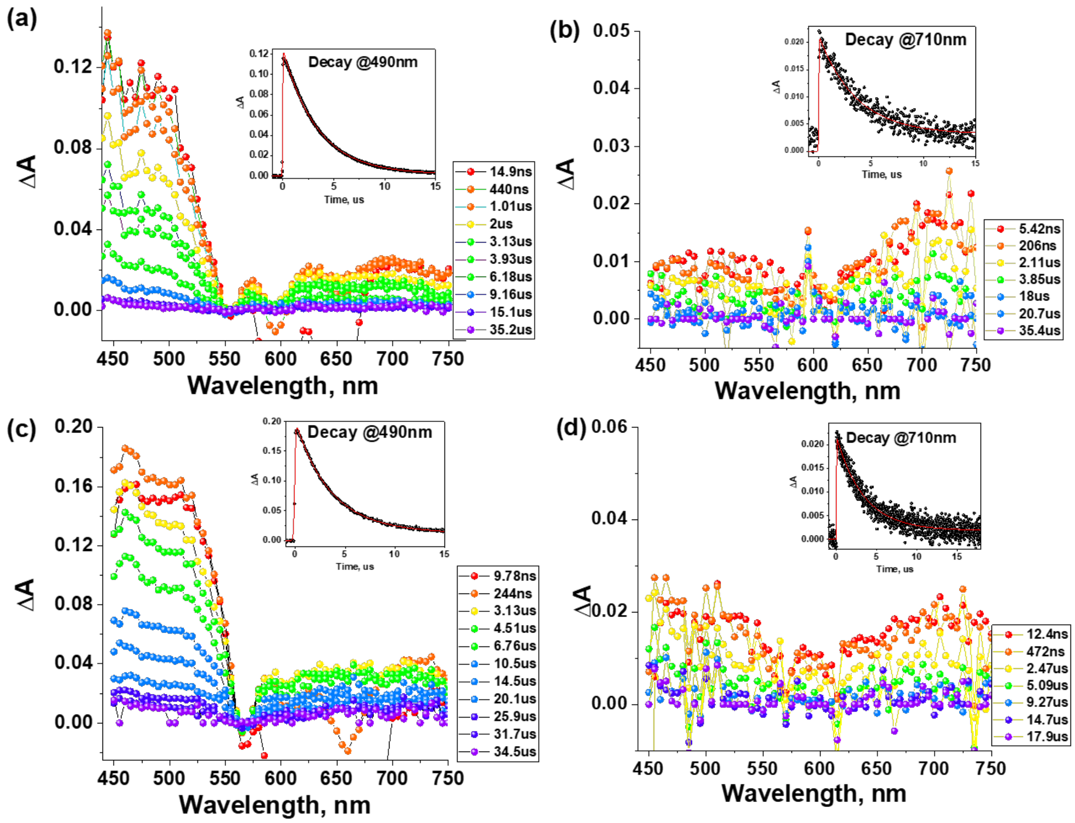

Figure S24. Nanosecond transient absorption spectra at indicated delay times (λ_{ex} =550 nm) of (a) SbP, (b) SbP-C_{60}, (c) $SbPF_3$, and (d, e) $SbPF_3$-C_{60} in CH_3CN. The inset shows the decay profile of the mentioned triplet peak.

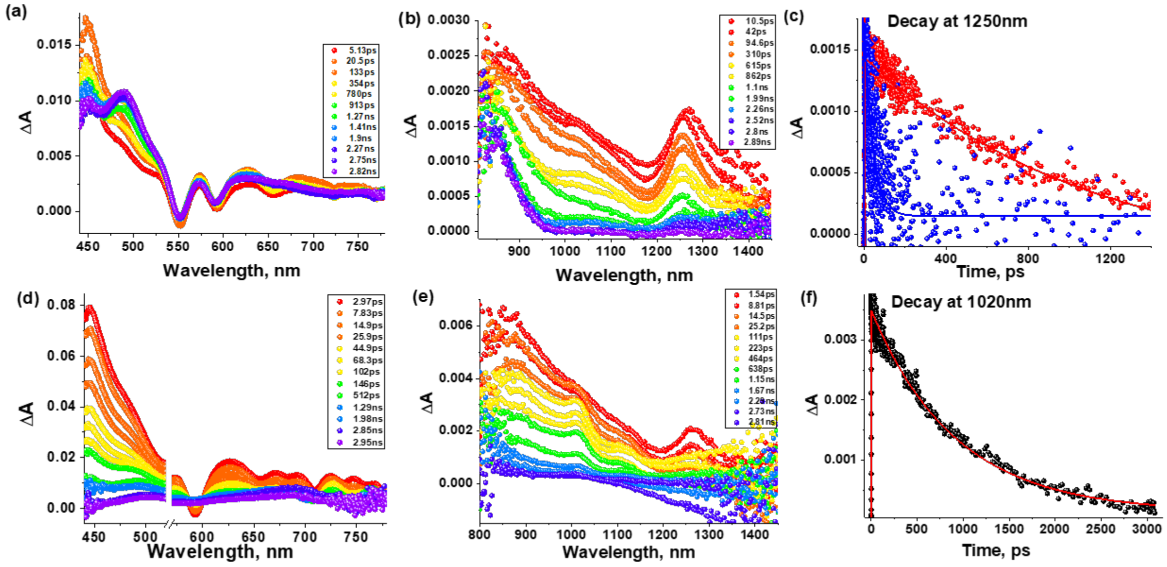

Figure S25. Femtosecond transient absorption spectra at indicated delay times (λ_{ex} =550nm) of (a, b) SbP (visible and NIR region) and (d, e) SbP-C_{60} (visible and NIR region) in toluene. Right panel (c) shows the time profile at 1250 nm of SbP (red) and conjugate SbP-C_{60} (blue) and (f) shows the decay profile at 1020 nm for the conjugate.

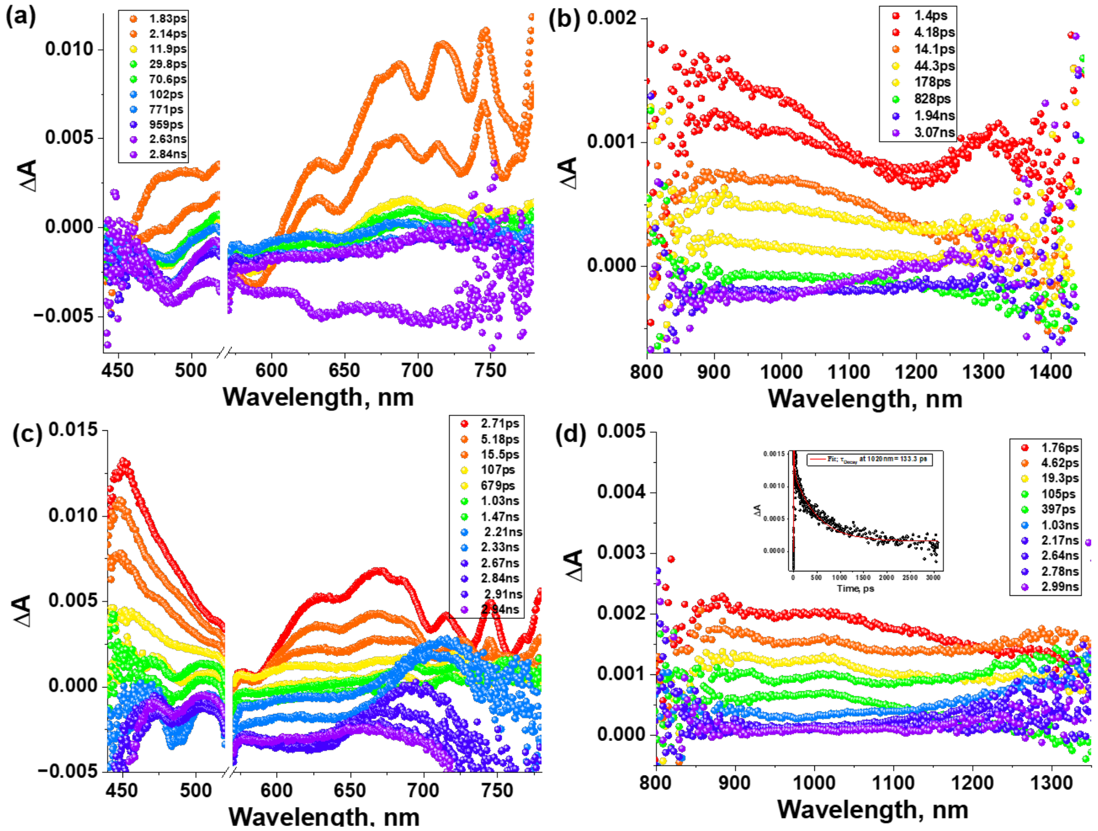

Figure S26. Femtosecond transient absorption spectra at indicated delay times (λ_{ex} = 550 nm) of (a, b) $SbPF_3$ (visible and NIR region) and (c, d) $SbPF_3$-C_{60} (visible and NIR region) in toluene inset shows the decay profile at 1020 nm for the conjugate.
